# An Ultra-High-Density, Transcript-Based, Genetic Map of Lettuce

**DOI:** 10.1534/g3.112.004929

**Published:** 2013-04-01

**Authors:** Maria José Truco, Hamid Ashrafi, Alexander Kozik, Hans van Leeuwen, John Bowers, Sebastian Reyes Chin Wo, Kevin Stoffel, Huaqin Xu, Theresa Hill, Allen Van Deynze, Richard W. Michelmore

**Affiliations:** * The Genome Center, University of California, Davis, California 95616; † Seed Biotechnology Center, University of California, Davis, California 95616; § Department of Plant Sciences, University of California, Davis, California 95616; ‡ Department of Plant Biology, University of Georgia, Athens, Georgia 30602

**Keywords:** *Lactuca sativa*, microarray, linkage analysis, recombination

## Abstract

We have generated an ultra-high-density genetic map for lettuce, an economically important member of the Compositae, consisting of 12,842 unigenes (13,943 markers) mapped in 3696 genetic bins distributed over nine chromosomal linkage groups. Genomic DNA was hybridized to a custom Affymetrix oligonucleotide array containing 6.4 million features representing 35,628 unigenes of *Lactuca* spp. Segregation of single-position polymorphisms was analyzed using 213 F_7:8_ recombinant inbred lines that had been generated by crossing cultivated *Lactuca sativa* cv. Salinas and *L. serriola* acc. US96UC23, the wild progenitor species of *L. sativa*. The high level of replication of each allele in the recombinant inbred lines was exploited to identify single-position polymorphisms that were assigned to parental haplotypes. Marker information has been made available using GBrowse to facilitate access to the map. This map has been anchored to the previously published integrated map of lettuce providing candidate genes for multiple phenotypes. The high density of markers achieved in this ultradense map allowed syntenic studies between lettuce and *Vitis vinifera* as well as other plant species.

Cultivated lettuce, *Lactuca sativa*, is extensively grown worldwide and is one of the most valuable crops in the US [$2.4 billion in 2011 (National Agricultural Statistics Service 2012)], as well as an important part of the American diet (United States Department of Agriculture 2011). It is a diploid (2n = 18) member of the Compositae with a genome size of 2.7 Gb (The Lettuce Genome Sequencing Consortium, unpublished data). *Lactuca serriola* is the likely wild progenitor of cultivated lettuce and a highly successful weedy species (Lindqvist 1960; Kesseli *et al.* 1991; de Vries 1997). *L. sativa* and *L. serriola* can be readily crossed, and there are no major barriers to gene flow between these species (Lindqvist 1960). *L. serriola* has served as a source of genetic variation for crop improvement, particularly disease-resistance genes (Crute 1988). A variety of genetic and genomic resources have been developed for lettuce as part of the Compositae Genome Project, including a consensus genetic map that comprises more than 2700 loci based on a variety of mainly anonymous molecular markers from several segregating populations (Truco *et al.* 2007) and extensive expressed sequence tag (EST) resources [http://compgenomics.ucdavis.edu (Stoffel *et al.* 2012)].

During the past 30 years, genetic maps for a broad variety of species have been generated with greater resolution and saturation as the numbers of molecular markers increased due to improvements in marker technologies. However, high-density maps have primarily relied on anonymous polymerase chain reaction−based markers such as random amplified polymorphic DNA, sequence-related amplified polymorphisms, and amplified fragment-length polymorphisms (*e.g.*, van Os *et al.* 2006; Troggio *et al.* 2007; Sun *et al.* 2007). Affymetrix microarrays provide a massively parallel technology for identification of polymorphism in genes. Thousands of loci can be genotyped in parallel by hybridizing DNA from each individual to an array comprised of millions of short oligonucleotide probes representing thousands of genes. Single-nucleotide polymorphisms (SNPs) or small insertion/deletions between the parents result in differences in the intensity of hybridization to the probes (Borevitz *et al.* 2003; West *et al.* 2006; Stoffel *et al.* 2012). Identification of segregating polymorphisms can be enhanced over parental comparisons by exploiting the high level of replication in recombinant inbred line (RIL) populations in which each individual is a mosaic of the two parental haplotypes (West *et al.* 2006).

Microarray data have been used to evaluate genetic diversity, construct genetic maps, and analyze the genetic architecture of traits. Borevitz *et al.* (2003) first demonstrated the detection of DNA sequence polymorphisms termed single-feature polymorphisms (SFPs) in a greater eukaryote that were subsequently used for studies of diversity in Arabidopsis (Borevitz *et al.* 2007). West *et al.* (2006) developed a genetic map with 599 SFPs to dissect expression of quantitative trait loci (QTL) and Singer *et al.* (2006) used an exon array for the generation of a high-resolution map comprising 676 genetic bins in Arabidopsis. In wheat, Bernardo *et al.* (2009) used an Affymetrix array to identify and map 877 SFPs by using 96 RILs and Banks *et al.* (2009) mapped 1035 SFPs by using a segregating population of doubled haploid lines. Edwards *et al.* (2008) used microarrays to analyze 880 insertion/deletion polymorphisms in rice and more recently Zhao *et al.* (2011) analyzed genetic variation in rice by using a 44,100 SNP array. Drost *et al.* (2009) constructed a genetic map of more than 600 markers by using an interspecific cross in *Populus* spp. and Neves *et al.* (2011) constructed a linkage map of 1845 genes in *Eucalyptus*. SFP-based maps also have led to identification of candidate genes and QTL (Werner *et al.* 2005; West *et al.* 2006).

Genetic maps have been constructed for lettuce using several intraspecific (Landry *et al.* 1987; Kesseli *et al.* 1994; Waycott *et al.* 1999; Hayashi *et al.* 2008) and interspecific populations (Johnson *et al.* 2000; Jeuken *et al.* 2001; Syed *et al.* 2006;) using a variety of molecular markers to provide increasingly dense maps as different technologies became available. Particular markers often only segregated in one or a few of these populations, which hampered the transfer of genetic and phenotypic information among the different maps. A single integrated map with 2744 loci was therefore constructed that combined seven separate maps into nine chromosomal linkage groups (Truco *et al.* 2007). Recently, genotyping by sequencing was used to generate a map of 1113 SNP markers (Truong *et al.* 2012). However, the majority of these loci in all of these maps are anonymous random amplified polymorphic DNA− and amplified fragment-length polymorphism−derived markers and therefore do not provide gene-based functional information. Only the map constructed by McHale *et al.* (2009) contains 772 EST sequence-based markers.

One of the populations used to produce the integrated map of lettuce was a RIL population resulting from an interspecific cross between *L. sativa* cv. Salinas × *L. serriola* acc. US96UC23. This population has emerged as a core reference mapping one and now comprises 356 F_7:8_ RILs. Sets of these RILs have been distributed to researchers worldwide for diverse studies. This population has also been genotyped for genes involved in disease resistance (McHale *et al.* 2009) and candidate genes for horticultural and domestication traits (Lavelle 2009) as well as phenotyped for multiple traits (Argyris *et al.* 2005, 2008; Zhang *et al.* 2007; Hartman *et al.* 2012). A large amount of genotypic and phenotypic information for this RIL population is available in the Compositae Genome Project database (http://compgenomics.ucdavis.edu).

In this article, we report the construction and characterization of an ultradense, transcript-based, genetic map of lettuce based on this intensively studied *L. sativa* cv. Salinas × *L. serriola* acc. US96UC23 RIL population. Genomic DNA samples from 213 RILs were hybridized to a custom Affymetrix GeneChip (Stoffel *et al.* 2012) to assay for polymorphisms in 35,628 unigenes derived from EST sequences of multiple *Lactuca* spp. Custom algorithms were used to identify single-position polymorphisms (SPPs) and to construct a genetic map composed of 12,841 unigenes in nine chromosomal linkage groups. This map provided a sufficient gene density for the identification of syntenic regions between lettuce and other plant species, such as *Vitis vinifera*, for which genome sequences were available.

## Materials and Methods

### Plant materials

A population of 213 F_7_ RILs from a cross between *L. sativa* cv. Salinas and *L. serriola* acc. US96UC23 (Johnson *et al.* 2000; Truco *et al.* 2007) was used to map SPPs. The cultivated parent, cv. Salinas (syn. Saladin), is a commercial crisphead cultivar that is widely grown in California and Europe and in the pedigree of many modern cultivars. Acc. US96UC23 is a wild accession of *L. serriola* that was collected in Davis, CA. *L. sativa* and *L. serriola* are closely related, have no barriers to hybridization, and may be con-specific (Lindqvist 1960; Kesseli *et al.* 1991; de Vries 1997). Care was taken during the generation of the RILs to minimize inadvertent selection and retain as much of the diversity in the population as possible; of the 375 F_2_ plants that were initially self-pollinated, 356 resulted in RILs, 213 of which were randomly selected for use in this study.

### DNA isolation, labeling, and microarray hybridization

Total genomic DNA was extracted from bulked leaf tissue from over 40 F_8_ plants from each of the 213 RILs and the parental lines and hybridized to the lettuce Affymetrix GeneChip following procedures described in Stoffel *et al.* (2012). To summarize, 30 µg of DNA from each RIL_7:8_ family and the two parental lines was sheared with DNase I to produce fragments between 50 and 250 bp in length, end-labeled, and hybridized to the lettuce GeneChip. Hybridizations to the 213 RIL DNA samples were carried out in duplicate, whereas the parental lines were each hybridized four times.

### Array design and data analysis

Details of the design of the lettuce GeneChip and data analysis are described in Stoffel *et al.* (2012). The lettuce GeneChip contains 6.4-million, 25 bp-long probes designed from 26,809 unigenes from cultivated lettuce (*L. sativa*) and an additional 8819 unique sequences from four other related *Lactuca* species (*L. serriola*, *L. saligna*, *L. virosa*, and *L. perennis*). This provided a total of 35,628 unigenes on the chip, of which 1184 were duplicated to assess the reproducibility of detecting polymorphisms (Stoffel *et al.* 2012); for the purpose of the initial analysis (algorithm training), duplicated unigenes were treated as separate markers. The lettuce GeneChip has an average of 187 probes per unigene that are tiled on alternating strands every 2 bp. A total of 21,920,928 transcribed bases are covered by the probes. Algorithms previously developed by West *et al.* (2006) were modified and a new software suite was developed to take advantage of the tiled design of the lettuce GeneChip that provides up to 13 overlapping probes at each position in a unigene covered by probes. We also took advantage of the redundancy that was provided by the genetic replication in the RIL population: hybridization intensity of each probe was measured 426 times corresponding to the 213 RILs replicated twice. This software suite for analyzing the lettuce GeneChip is available at http://chiplett.ucdavis.edu.

The raw data (CEL files) for hybridizations of 434 chips (2 × 213 RILs plus 4 replications of each parent) were obtained from Affymetrix GCOS (Affymetrix GeneChip Operating Software) v 1.3 software and subjected to robust multiarray analysis (Irizarry *et al.* 2003), background correction, and normalization using the Aroma Package of Bioconductor and R (http://www.braju.com/R/; http://www.r-project.org/). We subjected normalized data to quality control analysis by performing cluster analysis by using R on a subset of more than 20,000 probes that were considered informative based on a preliminary study. The two replications of each RIL clustered together for all but four RILs (~1% of all RILs); this lack of clustering between replicate hybridizations for these four RILs could have been the result of a variety of technical events and therefore another hybridization was performed for each of these RILs. Background correction, normalization, and QC analysis were repeated for chips of only those RILs that did not cluster together; in all cases the repeated chips clustered with one of the replicates and allowed the identification of the discordant chips, which were then removed from the original set of 434 chips and data from the replacement chips substituted. This provided a high confidence dataset of 434 chips on which a final background correction, normalization, and cluster analysis for all the data were performed.

### Detection of SPPs

The normalized data for ~6.4 million lettuce probes were analyzed using a custom Perl package (http://chiplett.ucdavis.edu/public/Manuals/manuals.php) to identify differences in hybridization in a sliding 2-bp window in regions of all unigenes covered by probes. Probes were assigned to GC bins based on their relative GC content and, within each GC bin, weighted mean hybridization intensity was calculated for each chip, hereafter called the reference set value. The hybridization intensity of each probe was weighted to take into consideration the distance of the central nucleotide of the probe to the position of the 2-bp window being interrogated using an empirically derived weighting curve that resulted in greater weight being given to central positions (Stoffel *et al.* 2012). The reference set value was calculated for all GC bins and all chips so that the data from each chip were comparable with a chip’s own reference set. A background signal for each chip was also calculated based on anti-genomic probes within each GC bin (Stoffel *et al.* 2012). A probe was excluded from further analysis if its hybridization intensity was less than the 90 percentile of the hybridization intensity of all anti-genomic probes (see Stoffel *et al.* 2012) in its specific GC bin. The weighted hybridization intensities were used to calculate the SPPdev at each 2-bp position covered by probes for all the chips using the equation:

$$\mathrm{SPPdev}=\left|\frac{\mathrm{Weighted}\;\mathrm{signal}\;\mathrm{intensity}\;\mathrm{of}\;\mathrm{probe}-\mathrm{Mean}\;\mathrm{intensity}\;\mathrm{reference}\;\mathrm{GC}\;\mathrm{bin}\;\mathrm{of}\;\mathrm{probe}}{\mathrm{Weighted}\;\mathrm{signal}\;\mathrm{intensity}\;\mathrm{of}\;\mathrm{probe}}\right|$$

For each 2-bp position, the SPPdev values for all the chips were then sorted from low to high. Our algorithm searched for a bimodal distribution using a user-defined difference (*e.g.*, 0.2). If the gap between two consecutive values was larger than the specified difference, an SPP was declared at that 2-bp position. If no bimodal distribution was found, the position was considered non-polymorphic and the next 2-bp position was analyzed. This was carried out iteratively for all positions on all contigs. A genotype was assigned to that 2-bp position based on the SPPdev values relative to the parental chips; the “A” genotype was assigned to alleles from cv. Salinas and the “B” genotype to alleles from acc. US96UC23. Both replicates of an individual RIL had to have the same genotype for an assignment to be made. If the replicates were inconsistent, the haplotype was treated as missing data. When contiguous 2-bp positions had bimodal distributions, they were summarized as one SPP range.

Summarized SPPs were filtered based on several criteria including minimum average SPPdev value, maximum percentage of missing data of a haplotype, minimum number of valid probes (those hybridizing to more than 90% of the anti-genomic probes), and minimum number of bases spanning an SPP. Consensus haplotypes were called from concatenated SPPs using a Python script (http://code.google.com/p/xuhu-rwm-map/source/browse/#svn/trunk). If the SPP calls within a unigene varied within a RIL, consistent with recombination within the unigene, the two most likely consensus haplotypes were calculated for these loci (http://code.google.com/p/xuhu-rwm-map/wiki/SplitSPPs).

### Genetic linkage analysis

Marker names were converted to four letter codes for simplicity and compatibility with mapping software; a conversion table for each four letter code and unigene ID is available at http://chiplett.ucdavis.edu/map_2012. SPPs were clustered into linkage groups using MadMapper (http://cgpdb.ucdavis.edu/XLinkage/MadMapper/) after filtering for missing data (genotype calls missing in no more than 25 RILs, 12%) and allelic distortion (the ratio between minor allele and major allele frequency had to be > 0.2). The linkage groups were assigned using previously mapped loci to ensure consistency with the published integrated map of lettuce (Truco *et al.* 2007). Pairwise recombination values among all markers were then calculated for each linkage group using Joinmap v.4 (Van Ooijen 2006). Markers with zero recombination among them were considered to belong to the same genetic bin (*e.g.*, Supporting Information, Figure S1). However, the assignment of markers into genetic bins was not always unequivocal due to missing data. Adjacent markers had to be assigned to a super-bin when there was no recombination for some combinations of markers but there was recombination between other markers within the same super-bin (*e.g.*, Table 1, Figure S2). The marker with the least missing data points was chosen to represent each bin or super-bin for genetic mapping purposes.

**Table 1:** Pairwise recombination values among markers in super-bin 36 in linkage group 4

Markers	AHPQ	AXWD	ASDC	AKIS	AXRS	ACAD
AHPQ	−	0	0	0	0	0.0024
AXWD		**−**	0	0	0	0.0024
ASDC			−	0	0	0.0025
AKIS				−	0.0026	0
AXRS					−	0.0026
ACAD						−

Markers representing each bin and super-bin, plus singletons (markers exhibiting recombination with all other markers in the linkage group) were mapped using RECORD_WIN (van Os *et al.* 2005), MSTMap (Wu *et al.* 2008), and Joinmap v4 (Van Ooijen 2006). RECORD_WIN was run with the settings: 30-cM gap size for all the linkage groups except LG4 and LG8 for which the gap size was 50 cM, Kosambi mapping function and 0.1 as the fraction of recombination allowed. MSTMap was run with the objective function COUNT to estimate map order and Kosambi mapping function to assign genetic distances. Due to the large number of markers segregating in the population, in Joinmap we used maximum likelihood mapping with the map order optimization parameters: chain length of 5000, initial acceptance probability 0.250, cooling control parameter of 0.001 stopping after 30,000 chains without improvement. For the estimation of recombination frequencies, we used 1000 for the length of burning-in chain, 4 for the number of EM cycles and 1000 for the chain length per EM cycle. Joinmap calculates genetic distances using Haldane’s mapping function which assumes no cross-over interference and therefore results in larger genetic maps than those generated using the Kosambi mapping function (Salomé *et al.* 2011). The marker orders generated with the three programs were examined using CheckMatrix (http://www.atgc.org/XLinkage/MadMapper/; http://code.google.com/p/atgc-map/) for inconsistencies.

Once the order of the unigenes was determined, regions of heterozygosity in individual RILs were identified by looking for contiguous regions with abnormally high rates of missing data compared with the same region in other RILs or high frequencies of apparent double cross-overs. Homozygous haplotypes were distinguished from heterozygous haplotypes using a sliding window of three markers (Figure S3). Heterozygous regions were defined as those where heterozygous haplotype windows were clustered.

Allelic segregation ratios were calculated for all unigenes. Goodness-of-fit χ^2^ tests were computed to the expected 1:1 Mendelian ratio. In each generation of selfing, the proportion of heterozygotes is reduced by half; therefore, the residual heterozygosity of an F_7_ RIL population is expected to be 1.56%. Because the RIL algorithm does not account for heterozygous alleles, this allelic class was not considered in the computations of goodness-of-fit. Markers were considered distorted at a level of significance of *P* < 0.05.

We estimated genome-wide linkage disequilibrium (LD) using the graphical genotyping program GTT 2.0 (Van Berloo 2008). To decrease the computational load, we analyzed 609 unigenes distributed at 5 cM intervals throughout the nine linkage groups. We used r^2^ as a measure of LD (Hill and Robertson 1968).

### Data curation

To facilitate data visualization, we generated a quasi-genomic sequence based on genetic distance and displayed the resultant chromosomal groups using GBrowse (http://www.Gbrowse.org). Each unigene was assigned coordinates based on the cumulative genetic distance from the beginning of the linkage group. Unigenes were separated by ‘N’s; the number of ‘N’s between markers was scaled to reflect the total number of cM (1585) and the physical size of the genome (2.7 Gb). Files were generated in GFF3 format and uploaded into GBrowse. For unigenes with two consensus haplotypes, both haplotypes were displayed if they mapped to different linkage groups (seven cases) or they were more than 5 cM apart in the same linkage group (four cases); otherwise, the position of only one of the haplotypes was displayed.

## Results

### Identification of SPPs

**Figure 1:**
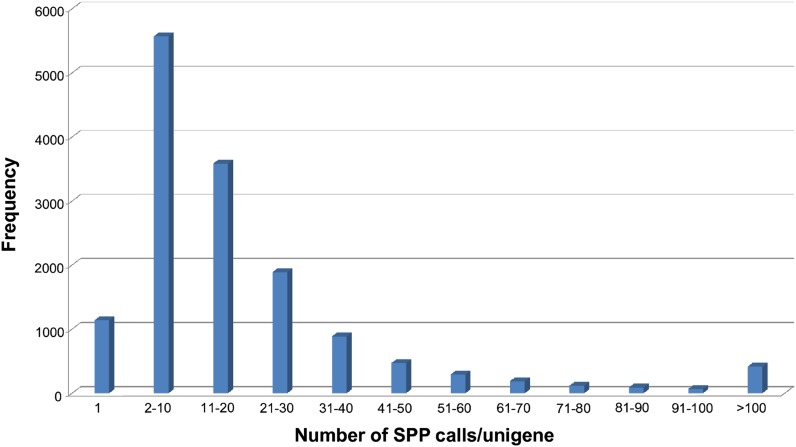
Frequency distribution of number of SPP calls per polymorphic unigene.

Analysis of hybridization values for the 213 RILs at all 2-bp windows covered by probes identified a total of 303,359 SPPs corresponding to 14,693 polymorphic loci of the 35,628 unigenes on the chip. This represents a frequency of 1 SPP per 72 bp assayed over a total of 21,920,928 bp of coding sequence addressed by the probes on the GeneChip. After consolidating contiguous SPPs into single consensus calls, we found that only 1137 polymorphic loci (7.7%) had a single SPP. Most polymorphic loci (13,124; 89.3%) had complete consensus of allele calls across the unigene within each RIL, the only differences among the RILs being due to missing data; single consensus haplotypes were designated for these loci. Therefore, the vast majority of the unigenes had a single haplotype in each RIL. However, a few loci (3%) varied in the allele calls for different SPPs within one or more RILs, which is consistent with recombination within the unigene; the two most likely consensus haplotypes were calculated for these unigenes accounting for 1569 additional loci. The average number of SPPs per polymorphic unigene was 20.6 with a median value of 12 (Figure 1). Loci with more than 25 (~8%) missing data points or with a minor allele frequency divided by major allele frequency of less than 0.2 (which equals to a minor allele frequency of 0.167) were not used for the initial genetic map construction. This resulted in a total of 13,788 polymorphic loci that were utilized for the initial mapping.

### Genetic map construction

**Figure 2:**
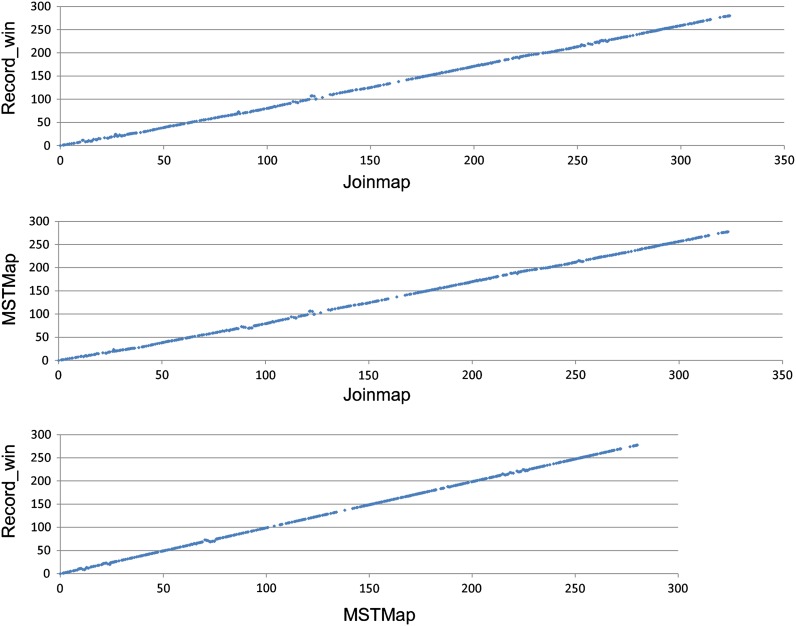
Dot plots of marker orders comparing the maps of LG1 generated by Joinmap, Record_Win, and MSTMap.

Approximately half of the loci (53.2%) grouped into super-bins, 27% grouped into bins, and 19.8% were singletons (Table 2). The average number of loci per super-bin was 9.18 with a median value of 7. The average number of loci per bin was 2.82 with a median value of 2. The genetic maps generated using three different mapping software programs (RECORD_Win, MSTMap, and Joinmap) were very similar. Discrepancies in order only occurred among very closely linked unigenes (Figure 2). The sizes of the linkage groups generated with RECORD_Win (2974 cM total) and MSTMap (2948 cM total) were very similar; Joinmap generated longer linkage groups (3483 cM total). None of the three maps was obviously superior to the others. We selected the genetic map generated using RECORD_Win as the reference map for the remainder of this study.

**Table 2:** Distribution of bins, super-bins and singletons by linkage group

LG	No. Markers	No. Bins (Markers)	No. Super-Bins (Markers)	No. Singletons	Size, cM
1	1481	140 (385)	81 (844)	252	144
2	1618	132 (384)	90 (921)	313	152
3	1415	143 (419)	80 (692)	304	174
4	1890	192 (518)	115 (989)	383	234
5	1998	202 (574)	117 (1026)	398	234
6	1217	120 (340)	71 (617)	260	142.5
7	1222	107 (303)	76 (711)	208	142
8	1959	183 (503)	115 (1045)	411	204
9	1143	114 (329)	66 (583)	231	158.5
Total	13,943	1333 (27%)	811 (53.2%)	2760 (19.8%)	1585

**Figure 3:**
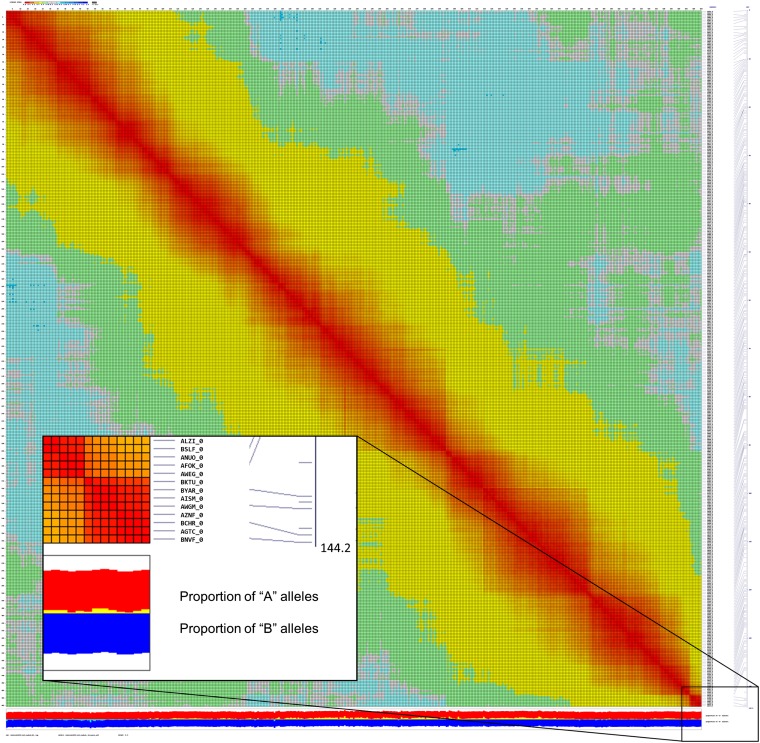
Heat map showing linkage along linkage group 1 displayed using Checkmatrix. Each chromosome is plotted in the linear order of the loci against itself and the degree of linkage between each locus indicated by a color. Red indicates <0.1 cM; yellow, 0.1−0.4 cM; green, 0.4−0.49 cM; blue, >0.51 cM. The first column at the right lists the locus name. The second column indicates the genetic distance. The red and blue bars at the bottom indicate the allelic composition at each locus. Red indicates Salinas allele; blue, *L. serriola* allele; yellow , distortion toward the Salinas allele; light blue, distortion toward *L. serriola* allele. Figures for each linkage group can be viewed at high magnification at http://chiplett.ucdavis.edu/map_2012. The insert in the bottom right corner illustrates a high magnification view of a subset of the data.

All 13,788 markers were initially grouped into 10 linkage groups using MadMapper. Because some unigenes had been previously mapped using Illumina GoldenGate SNP assays (http://cgpdb.ucdavis.edu/GeneticMapViewer/display/) (Truco *et al.* 2007; McHale *et al.* 2009), we were able to assign each of the linkage groups to the nine chromosomal linkage groups and orient them to be consistent with the published consensus integrated map (Table 2) (Truco *et al.* 2007). Markers were distributed throughout the linkage groups without any breaks (*e.g.*, Figure 3) except for linkage group 3. Linkage group A was provisionally assigned to linkage group 3 on the basis of markers that had been previously mapped in an interspecific *L. saligna* × *L. sativa* cross (Truco *et al.* 2007). Subsequent analysis using Record_Win joined LGA with LG3 separated by a gap of 21.3 cM.

Residual heterozygosity in an F_7_ RIL population is expected to be 1.56% (assuming a reduction of half in each generation of selfing). This would result in more than 46,000 heterozygous genotype calls for the 13,788 markers in the 213 RILs. Using a sliding window of three mapped markers (see *Materials and Methods*), we detected 1,031,346 windows indicative of heterozygosity indicating *ca*. 3% heterozygous positions. These regions of heterozygosity detected with the GeneChip were validated using SNPs previously mapped using Illumina GoldenGate Assays (McHale *et al.* 2009). The heterozygous regions detected using the two different technologies were concordant (Figure S4). More than 94% of the genome was heterozygous in at least one of the 213 RILs; 89% of the genome was heterozygous in two or more RILs.

The total map length generated by all three mapping programs was approximately twice or more the size of previous genetic maps constructed using the same population (RECORD_Win = 2,995 *vs.* 1,505 cM; Truco *et al.* 2007; McHale *et al.* 2009). Heterozygous regions resulted in abnormally high rates of missing data and/or high frequencies of apparent double cross-overs (single SPP in a RIL that differed from flanking loci; see *Materials and Methods*). Erroneously called double cross-overs would have artificially inflated genetic distances. To avoid this, we masked heterozygous regions by designating them as missing data and recalculated the marker order and genetic distances. This reduced the number of apparent double recombinants by 46% and the map size to 2247.5 cM. We then masked the remaining double recombinants because true double recombinants will be extremely rare with this marker density and again recalculated the gene order and genetic distances. These two revisions reduced the total map length to 1579 cM (48% reduction) with 13,788 markers and only *ca*. 4% missing data. This revised map is close to the total of 1505 cM observed in the previous studies (Truco *et al.* 2007; McHale *et al.* 2009). Map reduction occurred proportionally in all linkage groups. These recalculations occasionally changed the order of closely linked markers but marker order was generally maintained.

**Figure 4:**
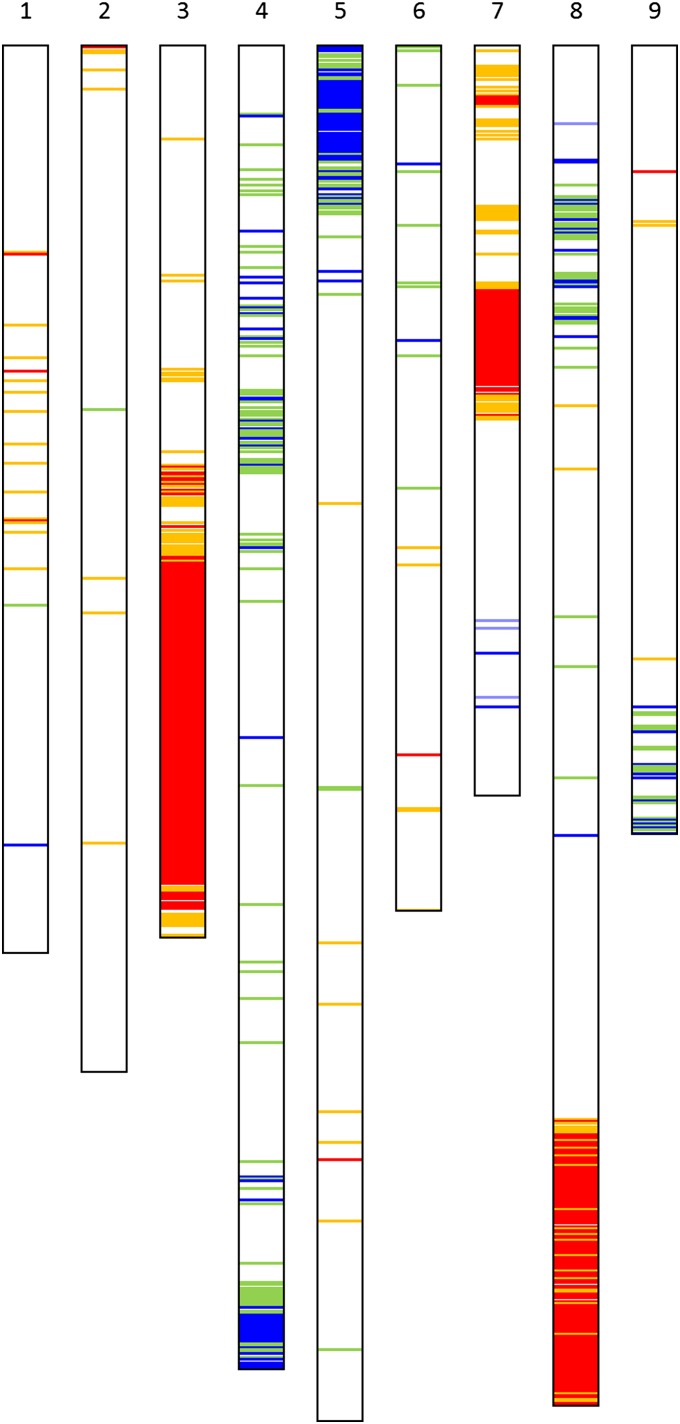
Distribution of 831 loci exhibiting distorted segregation on the nine chromosomes of lettuce. Orange and green lines indicate distortion at a significance level of *P* < 0.05. Red and blue lines indicate distortion at a significance level of *P* < 0.01. Distortions toward the *L. sativa* cv. Salinas and *L. serriola* alleles are in red/orange and blue/green, respectively.

Significant segregation distortion (*P* < 0.05) from the expected segregation ratio of 1:1 indicative of unintended selection during the generation of the RILs (*e.g.*, Salomé *et al.* 2011) was observed in all linkage groups (Figure 4). Regions of linkage groups 3, 4, 5, 7, and 8 were significantly distorted (*P* < 0.01). The distortions on linkage groups 3, 7, and bottom part of 8 were toward an excess of the *L. sativa* allele. On linkage groups 4, 5, top 8, and 9 the distortions were toward an excess of the *L. serriola* allele (Figure 4). Overall, segregation distortion slightly favored alleles from the domesticated parent over those from the wild parent (11 *vs.* 6% respectively).

A region of distortion (*P* < 0.01) toward the Salinas allele in LG3 (see lower region of LG3 in Figure 4) included the gap of 21.3 cM in the initial map (see above). Three hundred and forty-three markers had been excluded from the initial mapping analysis because the frequency of the minor allele was below the threshold of ~17% (see *Materials and Methods*). Of these skewed loci, 261 were distorted toward the Salinas allele. We conducted a further round of grouping with MadMapper using all loci that mapped in LG3 plus all those loci that had been previously excluded with distortion toward the Salinas allele. One hundred fifty-five skewed loci grouped with LG3 and after a new round of mapping using Record_Win were linked inside and around the 21.3 cM gap, filling it completely. The final corrected map has 1585 cM and 13,943 loci corresponding to 12,842 unigenes distributed in nine linkage groups corresponding to the nine chromosomes of lettuce (Table 2). Raw data for the map construction and maps of all the linkage groups can be viewed at http://chiplett.ucdavis.edu/map_2012.

To search for potential epistatic interactions resulting in unexpected combinations of unlinked loci (Wade *et al.* 2001; Isobe *et al.* 2012), we estimated genome-wide LD for 609 unigenes distributed approximately every 5 cM throughout the genome. No r^2^ values greater than 0.5 were observed among pairs of loci that mapped to different linkage groups. Greater r^2^ values were only observed among loci mapping together in the same linkage group. Therefore, there was no evidence for particular combinations of alleles at loci in different chromosomes being selected for or against during the generation of the RILs.

**Figure 5:**
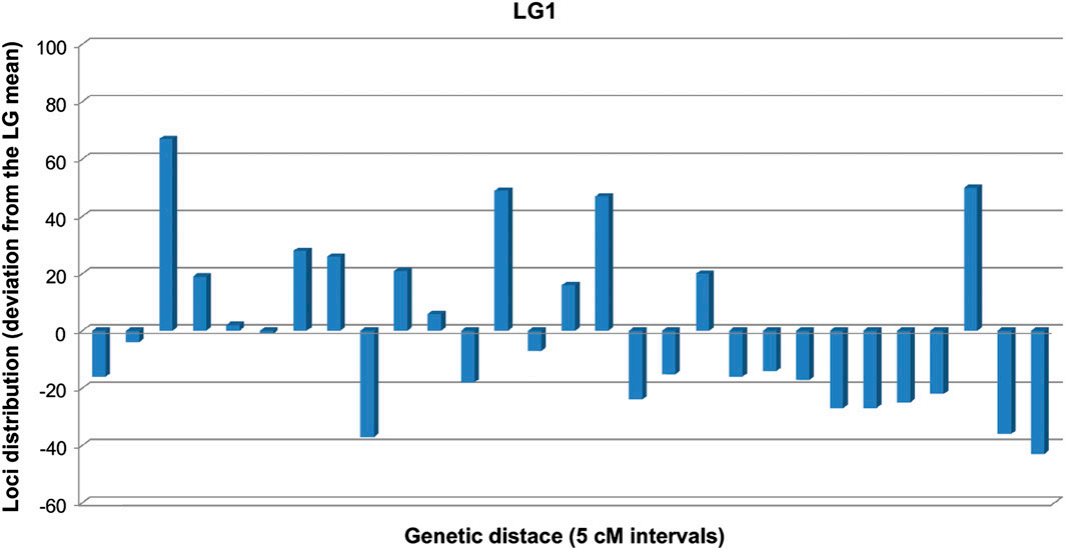
Average distribution of loci in intervals of 5 cM along LG1. Deviation from the mean value for LG1 (x = 51) is shown by bars. Similar analyses for the other LGs are shown in Figure S5.

The distribution of polymorphic loci with respect to genetic distance was analyzed for each linkage group. We determined the number of polymorphic loci within each 5 cM interval along the total length of each linkage group. The distribution of number of loci in each interval was compared to the Poisson distribution (P(x) = e^-µ^µ^x^/x!, where µ is the average number of loci per interval over the entire map and x in the actual number of loci) expected if loci were distributed randomly. Observed and expected frequencies were compared using a χ^2^ test (Troggio *et al.* 2007). Polymorphic loci were not randomly distributed along the linkage groups (Figure 5). The distribution of loci was different for each linkage group (Figure S5). Linkage groups 1 and 2 had a greater average number of loci and the greatest deviation from their respective means.

### Validation of SPPs using Illumina GoldenGate SNP assays

To validate the SPP allele calls we used data for unigenes present on the lettuce Affymetrix GeneChip that had also been mapped on 188 RILs using Illumina GoldenGate Assays (McHale *et al.* 2009). Allele calls were compared individually for 96 unigenes distributed throughout the nine chromosomal linkage groups. The allelic calls for 95 of the 96 unigenes were 99.9% identical; of 17,860 comparisons between the two marker technologies (188 RILs × 95 unigenes), there were only 22 (0.1%) discordant allelic calls. The remaining unigene, *LSRGCAE05-1*, encodes a NBS-LRR candidate disease resistance protein (Genbank number AY153843) and nearly 50% of its allele calls were discordant between the two technologies. Resistance genes are frequently members of multi-gene families (Michelmore and Myers 1998). Detection of gene assignment is likely to be problematic for such highly polymorphic multigene families with both of these technologies (Hill *et al.* 2012); it is possible that each technology detected polymorphism among paralogs located at different chromosomal positions.

### Distribution of recombination events

**Figure 6:**
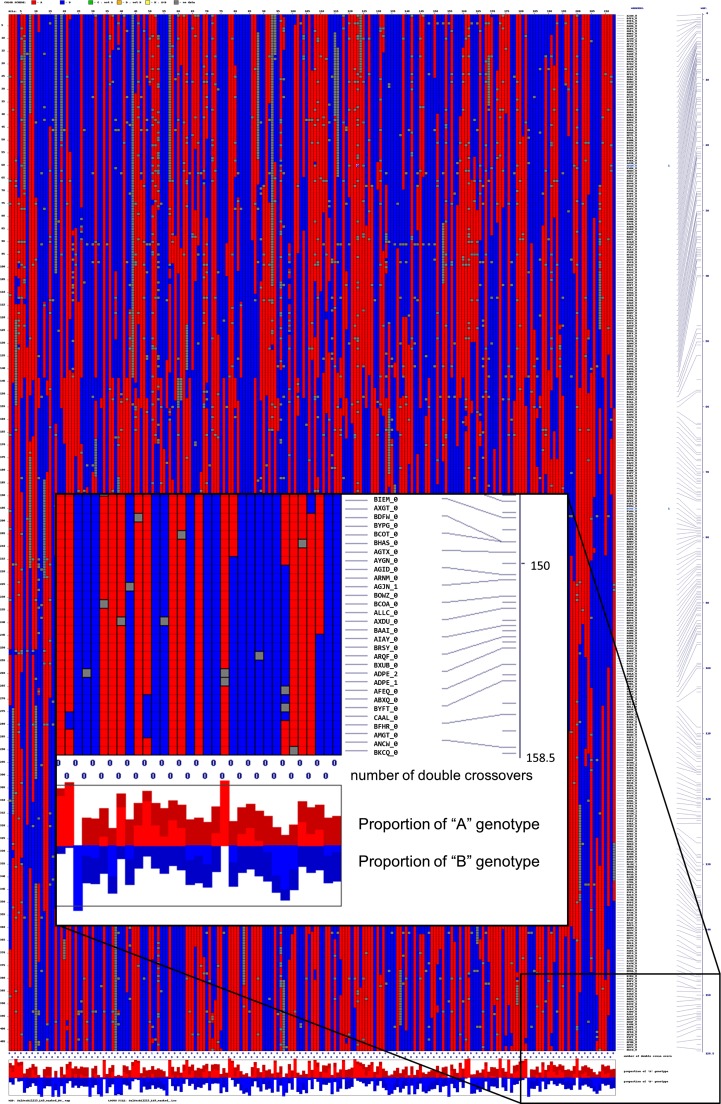
Distribution of haplotypes along linkage group nine displayed vertically for 213 RILs using Checkmatrix. The RIL families are arranged along the x-axis and the loci displayed in linear order along the y-axis. Red indicates *L. sativa* haplotype; blue, *L. serriola* haplotype. Gray, no allele called; missing data. The first column at the right lists the locus name. The second column indicates the genetic distance. The number of double cross-overs and the proportions of each haplotype are shown below each RIL. Displays for each linkage group can be viewed at high magnification at http://chiplett.ucdavis.edu/map_2012. The insert in the bottom right corner illustrates a high magnification view of a subset of the data.

**Figure 7:**
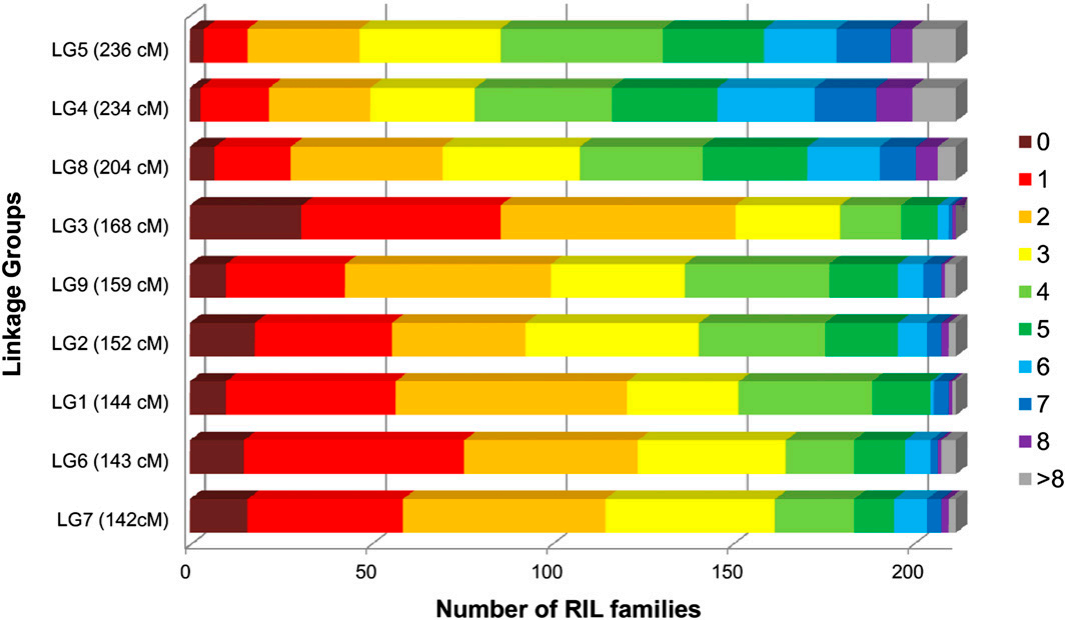
Frequency distribution of the number of cross-overs. Numbers of cross-overs detected per RIL (0 to >8) are displayed chromatically for each linkage group, which are organized by genetic size (longest at top, shortest at bottom).

The high density of markers mapped in this study allowed the identification of nearly all recombination events present in each RIL (*e.g.*, Figure 6). We calculated the number of cross-overs per RIL using markers spaced every 5 cM; this varied from 10 to 56 with a mean of 28.8. This is close to the value of 35 that is expected assuming one cross-over per chromosome arm per meiosis and a reduction each generation of selfing in the ability to detect cross-overs by half due to homozygosity. The number of cross-overs detected per chromosome ranged from 0 to more than 8; the number of cross-overs was correlated with genetic size (Figure 7). Chromosomes with no apparent cross-overs were found for all the linkage groups but were more frequent for the smaller linkage groups. We performed a QTL analysis of cross-over frequency to determine whether QTL influencing recombination rate were segregating in this population; however, we did not detect any such QTL.

### Chromosomal distribution of diversity

**Figure 8:**
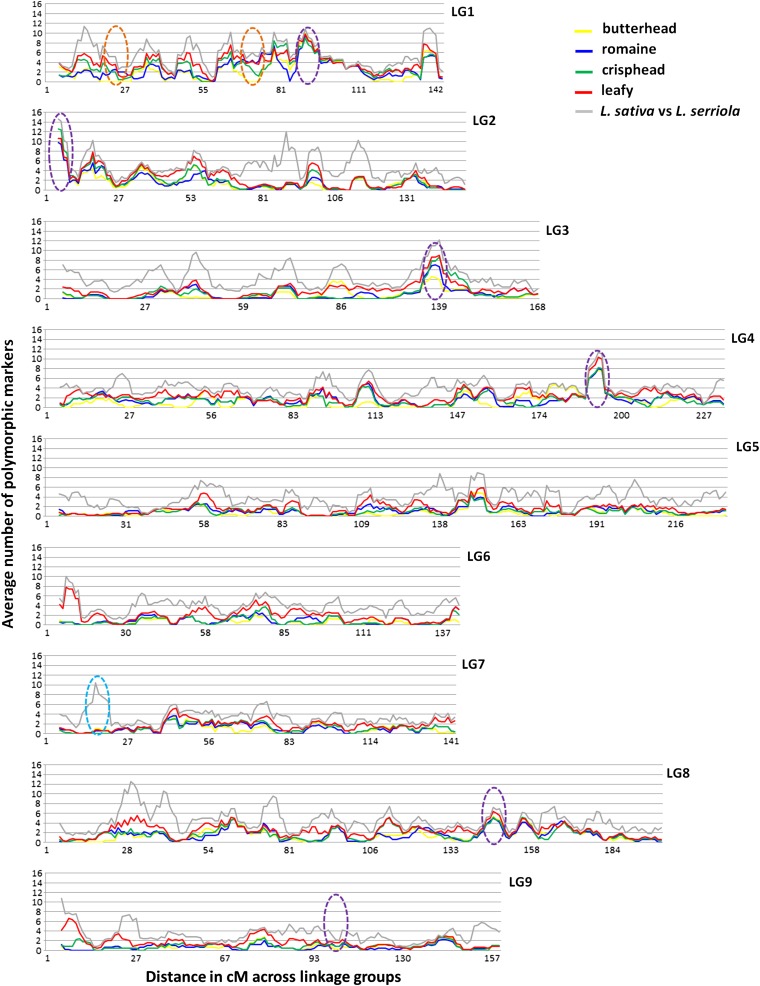
Distribution of haplotype diversity across the nine linkage groups for butterhead (yellow line), romaine (blue line), crisphead (green line), and leafy (red line) lettuce types and between *L. sativa* and *L. serriola* (gray line). Each y-axis indicates the average number of polymorphic loci in a sliding 5-cM window (from 0 to 16). Turquoise ellipse: example of region with low level of diversity in cultivated types relative to *L. serriola*. Orange ellipses: examples of regions with low diversity specific to one type, in this case butterhead. Purple ellipses: regions associated with clusters of NBS-LRR-encoding genes.

We assessed the distribution of genetic diversity along each linkage group by using a panel of 52 accessions that was composed mostly of *L. sativa* cultivars encompassing diversity in each of the cultivated types of lettuce (Table S1) (Stoffel *et al.* 2012) and 10,243 unigenes distributed throughout the nine linkage groups. The average number of haplotypes detected per unigene across all the linkage groups was 3.4 with a median of 3.0 and a range from 2 to 22. We estimated diversity by summing the number of haplotypes detected in the diversity panel over each cM and averaging the values in sliding windows of 5 cM. Regions of high or low diversity were detected in all LGs (Figure 8). Across most of the genome the pattern of diversity was remarkably parallel for all lettuce types with *L. serriola* being the most diverse as expected. In a few regions, *e.g.*, on LG7, the level of diversity in the cultivated types was much lower than in the *L. serriola*, indicating a loss of diversity during domestication (turquoise ellipse; Figure 8). Some regions of low diversity were specific to a particular type, *e.g.*, butterhead types had two regions of low diversity in LG1 (orange ellipses; Figure 8). Five regions with high levels of diversity in all types of lettuce similar to the level of polymorphism observed in *L. serriola* corresponded to five clusters of NBS-LRR-encoding genes responsible for pathogen recognition that had been mapped previously in LG1, 2, 3b, 4, and 8; only the cluster of NBS-LRR-encoding genes that did not show high levels of polymorphism mapped to LG9 (purple ellipses, Figure 8) (McHale *et al.*, 2009).

### Map curation

To provide convenient access to the unigenes on the ultradense map of lettuce, a quasi-genomic sequence of lettuce was generated based on genetic distances and visualized using GBrowse (http://lgr.genomecenter.ucdavis.edu). The 13,943 mapped markers were concatenated in their genetic order for each of the nine linkage groups. Markers within a bin were ordered randomly. Bins were separated by ‘N’s scaled to reflect genetic distances between them with 1 cM represented by 1730 kb for a total of 1585 cM spanning 2.7 Gb. Unigenes were assigned coordinates and displayed relative to information for each unigene such as SNPs, SPPs and the sequences of the GeneChip probes.

### Syntenic analyses

**Figure 9:**
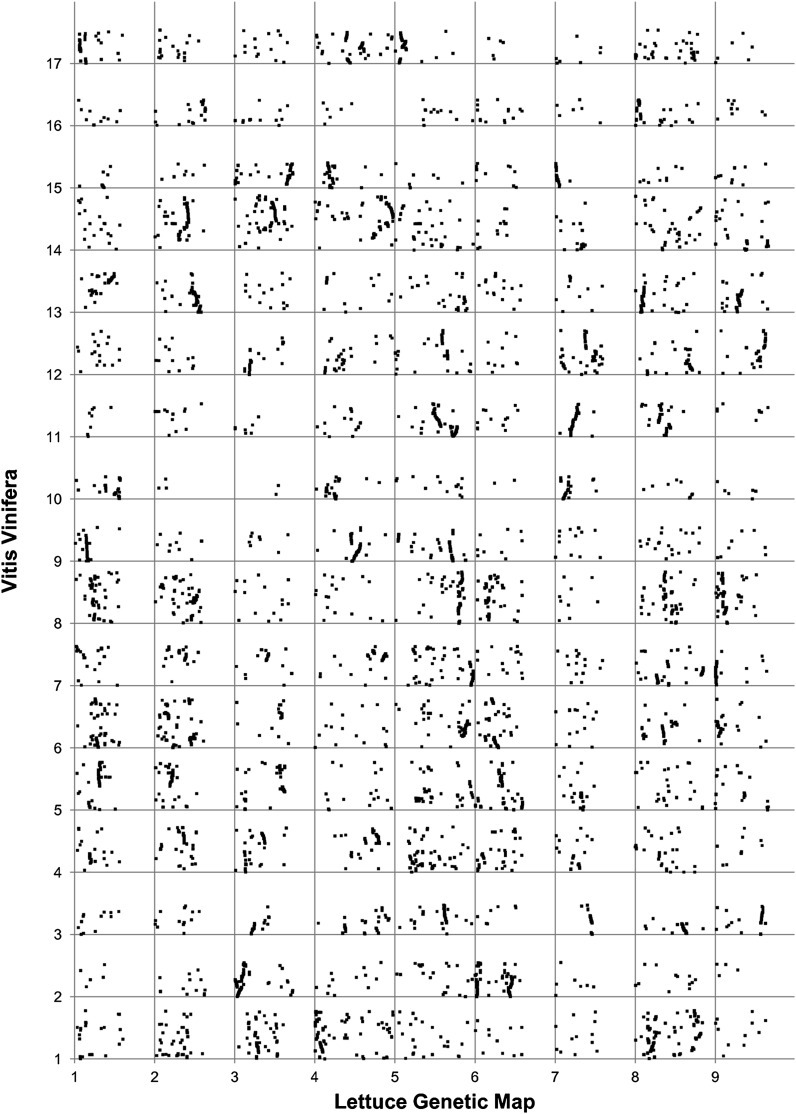
Dot plot of the syntenic positions of genes in grape and lettuce. The nine chromosomes of lettuce are ordered along the x-axis and the 17 chromosomes of grape along the y-axis. The positions of 10,552 loci mapped in lettuce on the grape genome are displayed.

We analyzed synteny between lettuce and sequenced eudicots including grape (*Vitis vinifera*), poplar (*Populus trichocarpa*), Arabidopsis (*A. thaliana*), and potato (*Solanum tuberosum*). The 13,788 loci from the lettuce map were compared to genes predicted in the 12x assembly of grape (http://www.genoscope.cns.fr/externe/Download/Projets/Projet_ML/data/12X/assembly/), a revision of the 8x published assembly by Jaillon *et al.* (2007). Similar genes were identified as the best TBLASTX hit with e < 1 × e^-6^. Of the 12,658 mapped lettuce unigenes, 10,552 matched chromosomally located genes of grape and 564 matched genes that were on non-chromosomally assigned assemblies of the grape genome. Many small regions of synteny were identified (Figure 9). Each of 38 chromosomal arms of grape is present three times in lettuce. This has resulted in *ca*. 114 major segments of synteny between the two species, approximately 11 per lettuce chromosome. Lettuce is, therefore, an ancient hexaploid compared with grape. In contrast, each region of the lettuce genome matched only one region in the grape genome indicating that grape has not undergone additional polyploidy events since its last common ancestor with lettuce. The gene order for some complete chromosomes of grape has been maintained intact in lettuce, whereas for other chromosomes only chromosomal arms have been maintained intact. Comparisons with poplar, Arabidopsis, and potato (data not shown) revealed more complex patterns of synteny because these species have undergone one or more additional polyploidy events in their lineages since their last common ancestor with lettuce. Arabidopsis has undergone two additional cycles of polyploidy followed by differential gene loss, along with a relatively large number of chromosomal fusions, translocations and inversions (Yogeeswaran *et al.* 2005; Lysak *et al.* 2006; Hu *et al.* 2011), with the result that remaining segments of synteny are far shorter and more complex than those observed in the comparison to grape.

## Discussion

The genetic map described in this article represents the most dense map developed for lettuce to date and will be the foundation for fine mapping of quantitative trait loci, identifying candidate genes, map-based cloning and genome assembly efforts, as well as marker-assisted selection in breeding programs. The initial map that we constructed was based on 13,788 loci that spanned 2,974 cM. This was nearly double the size of the previously published consensus map that was developed from seven intra- and interspecific mapping populations using a total of 560 markers that segregated in two or more populations (Truco *et al.* 2007). Genotyping errors can cause increases in genetic map sizes; every 1% error rate in a marker adds *ca*. 2 cM to the map (Cartwright *et al.* 2007). By masking heterozygous regions and removing double-crossover events, and adding a few heavily skewed loci the total map distance was reduced to 1585 cM, close to the previously observed value of 1505 cM for this population (Truco *et al.* 2007). A small increase in size was also observed in a SFP-based linkage map for Arabidopsis as compared to maps generated previously with fewer markers [from 397.1 to 422.5 cM (Singer *et al.* 2006) or to 625 cM (Truong *et al.* 2012)].

The analysis of 213 RILS with 13,943 SPP markers provided a highly accurate map in which most of the recombination events in each of the RILs were detected. SPP marker calls were validated by their consistency within unigenes and genetic bins as well as concordance using a different SNP marker technology. The similarity of the maps generated with three different mapping programs provides further support for the quality of the map. Recently the IN algorithm that is a part of RECORD was reported to be the most accurate for estimating gene orders in dense maps (Wu *et al.* 2011). This finding is consistent with high levels of polymorphism segregating in this wild by domesticate population and no regions having identity by descent.

This map is one of the densest meiotic maps for any eukaryotic species. With more than 10,000 markers, it is the first ultradense map for a member of the Compositae and complements the recent 10,000 marker map for sunflower (Bowers *et al.* 2012). Although genetic maps with several thousand markers are available for a broad range of plant and animal species, few maps have more than 5000 markers, and most of the dense maps have been based on anonymous, polymerase chain reaction−based markers (reviewed in van Os *et al.* 2006), although the situation is changing rapidly with the advent of genotyping by sequencing (reviewed in Davey *et al.* 2011). In contrast to these maps, our map of lettuce is based on 12,842 transcribed unigenes and therefore has much greater information value.

Assuming lettuce has *ca*. 45,000 genes (Lettuce Genome Sequencing Consortium, unpublished data), this map provides the genetic locations of approximately one quarter to one third of the protein-encoding genes expressed in lettuce. Given that the GeneChip detects polymorphisms in ESTs, this map captures a large fraction of the genes determining the phenotypic differences segregating in this population. Details of the mapped genes are provided through a GBrowse-based web browser (http://lgr.genomecenter.ucdavis.edu). Ultradense maps avoid the necessity for separate bulked segregant analyses to saturate individual target regions (Michelmore *et al.* 1991). High densities of markers also allow chromosome landing (Tanksley *et al.* 1995) rather than walking using bacterial artificial chromosomes or genome assemblies for map-based cloning. Given a lettuce genome size of 2.7 Gb (The Lettuce Genome Sequencing Consortium, unpublished data), there is a marker every *ca*. 200 kb on average, although the relationship between physical and genetic distances varies greatly (Fu *et al.* 2002; Sourdille *et al.* 2004; Wang *et al.* 2011). The average genetic distance between markers (bins) was 0.4 cM. The analysis of 213 RILs (representing >2100 meioses) provided a genetic resolution of less than three mapped genes on average per genetic bin facilitating the identification of candidate genes. The analysis of such a large number of RILs also had the advantage in that more than 94% of the genome was heterozygous in at least one RIL. This provides suitable starting material for fine mapping genes by saturating target regions with recombinants using flanking heterozygous markers with minimal genetic noise from other regions of the genome.

The genetic structure of this RIL population is similar to that observed in RIL populations of other species. Although this map was generated from a wild by domesticate cross, there was only limited segregation distortion and no major blocks of repressed recombination indicative of structural rearrangements. This provides further support for the conspecific relationship of *L. sativa* and *L. serriola* and the domestication of cultivated lettuce from the weedy *L. serriola* (Lindqvist 1960; Kesseli *et al.* 1991; de Vries 1997). The clustering of loci exhibiting segregation distortion suggests that the distortion has a genetic basis rather than being an experimental artifact. Seventeen percent of the genome exhibited segregation distortion with 11% and 6% elevated for the domesticated and wild haplotypes respectively. However, segregation distortion was never absolute; all haplotypes were recovered, albeit sometimes infrequently. The regions exhibiting segregation distortion detected here are similar to those detected in other lettuce populations (Truco *et al.* 2007; Uwimana *et al.* 2012). Segregation distortion can be due to several reasons, including preferential gametic transmission (Moyle and Graham 2006; Li *et al.* 2010), genetic elements that enhance their own transmission (Rebollo *et al.* 2012), inadvertent selection due to differences in viability and fitness over multiple generations during RIL construction due to one or the other allele of a specific gene conferring a selective advantage (Salomé *et al.* 2011), and the effects of deleterious allelic combinations generated during inbreeding (MacDaniel and Shaw 2007; Salomé *et al.* 2011). Considerable care was taken to minimize selection during the generation of RILs used in this study. Also, there was no evidence for deleterious allelic combinations at unlinked loci.

Syntenic comparisons of sequenced plant genomes provide insights into the evolutionary events that shaped the lettuce genome as well as allow inferences as to the positions of genes that have yet to be mapped. Our data on the triplication of the lettuce genome relative to *Vitis vinifera* is indicative of two genome duplication events in the Compositae lineage since its divergence from *V. vinifera* and confirms the inference based on EST analyses (Barker *et al.* 2008). The same result was observed in tomato, which is in the Asterid I group *vs.* the Asterid II group, which contains lettuce (The Tomato Genome Consortium 2012); this finding suggests that the triplication event occurred before the radiation of the Asterids but after their divergence from the lineage that gave rise to *V. vinifera*. Extensive rearrangements and only short regions of synteny were expected because *Lactuca* spp. are rapid cycling, highly successful weeds. This is similar to Arabidopsis and the *Brassica* species relative to species such as *V. vinifera* and poplar that reproduce clonally and have longer sexual generation times (Yogeeswaran *et al.* 2005; Lysak *et al.* 2006; Hu *et al.* 2011).

### Future directions

This map provides many polymorphic candidate genes for map-based cloning. Several studies are in progress to clone and characterize genes involved in domestication traits such as branching, spininess, leaf shape, seed physiology, and flowering time. In addition, the lettuce genome has recently been sequenced and annotation is underway (Lettuce Genome Consortium, unpublished data); this map provides the opportunity to validate and refine the genome assemblies and place them in chromosomal linkage groups.

The same lettuce GeneChip used to produce this ultradense map has been used for studies of diversity in 52 genotypes of cultivated lettuce (*L. sativa*) and several wild relatives of lettuce (Stoffel *et al.* 2012). Coupling the ultradense genetic map with the diversity data will allow the deployment of the information generated in a single population to other populations for which the parental lines were included in the diversity panel, which will facilitate the identification of polymorphic markers in regions of interest and further assist in the identification of candidate genes for traits of scientific interest and/or agronomic importance (Argyris *et al.* 2010).

The ultradense map reported here was based on SPPs detected using a custom microarray. However, microarray technology is being superseded by genotyping by sequencing the whole or reduced components of the genome (Huang *et al.* 2009; Xie *et al.* 2010; Elshire *et al.* 2011; Davey *et al.* 2011; Poland *et al.* 2012). The gene space of 96 RILs from the same *L. sativa* x *L. serriola* population is being sequenced and the data from the lettuce GeneChip will be used to validate the mapping of the SNPs detected by sequencing.

## Supplementary Material

Supporting Information
