# Supplementary material for: An Ultra-High-Density, Transcript-Based, Genetic Map of Lettuce
Source: G3 (Bethesda). 2013 Apr 1;3(4):617–31. doi: 10.1534/g3.112.004929 (PMC3618349; doi:10.1534/g3.112.004929)
Supplement: Supporting Information [file supp_3_4_617__index.html]

An Ultra High-Density, Transcript-Based, Genetic Map of Lettuce — An Ultra High-Density, Transcript-Based, Genetic Map of Lettuce — An Ultra-High-Density, Transcript-Based, Genetic Map of Lettuce — Supporting Information 

# An Ultra-High-Density, Transcript-Based, Genetic Map of Lettuce

## Supporting Information for Truco *et al.*, 2013

**Files in this Data Supplement:**

- Supporting Information - Figures S1-S5 and Table S1 (PDF, 2 MB)
- Figure S1 - Haplotypes of all RIL families for the six molecular markers grouped in bin2 in linkage group 4 (PDF, 835 KB)
- Figure S2 - Haplotypes of all RIL families for the six molecular markers grouped in sbin36 in linkage group 4 (PDF, 891 KB)
- Figure S3 - Homozygous or heterozygous haplotypes identified using a sliding window of three markers (PDF, 136 KB)
- Figure S4 - Correspondence of heterozygous regions of LG1 in three RILs (#19, 21, and 104) estimated using the GeneChip (left) and using Illumina Golden Gate SNP markers (right) (PDF, 152 KB)
- Figure S5 - Average distribution of loci in intervals of 5 cM along LG2 to LG9 (PDF, 159 KB)
- Table S1 - The 52 accessions of *L. sativa* and *L. serriola* used to assess the distribution of genetic diversity along each linkage group (PDF, 74 KB)
