# Supplementary material for: An Ultra-High-Density, Transcript-Based, Genetic Map of Lettuce
Source: G3 (Bethesda). 2013 Apr 1;3(4):617–31. doi: 10.1534/g3.112.004929 (PMC3618349; doi:10.1534/g3.112.004929)
Supplement: Supporting Information [file supp_g3.112.004929_004929SI.pdf]

G3 Genes | Genomes | Genetics

An Ultra High-Density, Transcript-Based, Genetic Map of Lettuce

Maria José Truco,[*] Hamid Ashrafi,[§] Alexander Kozik,[*] Hans van Leeuwen,[*,1] John Bowers,[**] Sebastian Reyes Chin Wo,[*] Kevin Stoffel,[§] Huaqin Xu,[*] Theresa Hill,[§] Allen Van Deynze,[§,§§] and Richard W. Michelmore[*,§§,2]

[*]The Genome Center, [§]Seed Biotechnology Center, and [§§]Department of Plant Sciences, University of California, Davis, California 95616, and [**]Department of Plant Biology, University of Georgia, Athens, Georgia 30602

[1] Current address: Nunhems Netherlands B.V., 6080 AA Haelen, The Netherlands

[2]Corresponding author: The Genome Center, One Shields Avenue, The University of California, Davis, CA 95616. E-mail: rwmichelmore@ucdavis.edu

DOI: 10.1534/g3.112.004929

RIL families	1	2	3	4	5	6	7	8	9	11	12	13	14	15	16	17	18	19	20	21	22	23	24	25	26	27	29	30	31	32	33	34	35	36	37	39	40	41	42	43	44	45	47	48	50	51	52	53	55	56
AAFL_6_1	B	B	B	A	A	A	B	B	B	B	A	B	B	A	A	B	B	B	B	B	A	A	A	B	B	A	A	B	A	B	A	A	B	A	A	A	A	B	A	A	A	A	B	B	A	A	A	A	B	A
AKEZ_2_0	B	B	B	A	A	A	B	B	B	B	A	B	B	A	A	B	B	B	B	B	A	A	A	B	B	A	A	B	A	B	A	A	B	A	A	A	A	B	A	A	A	A	B	B	A	A	A	A	B	A
AQER_183_0	B	B	B	A	A	A	B	B	B	B	A	B	B	A	A	B	B	B	B	B	A	A	A	B	B	A	A	B	A	B	A	A	B	A	A	A	A	B	A	A	A	A	B	B	A	A	A	A	B	A
ATMV_35_0	B	B	B	A	A	A	B	B	B	B	A	B	B	A	-	B	B	B	B	B	A	A	A	B	B	A	A	B	A	B	A	A	B	A	A	A	A	B	A	A	A	A	B	B	A	A	A	A	B	A
BGOM_16_0	B	B	B	A	A	A	B	B	B	B	A	B	B	A	A	B	B	B	B	B	-	A	A	B	B	A	A	B	A	B	A	A	B	A	A	A	A	B	A	A	A	A	B	B	A	A	A	A	B	A
BGST_51_0	B	B	B	A	A	A	B	B	B	B	A	B	B	A	A	B	B	B	B	B	A	A	A	B	B	A	A	B	A	B	A	A	B	A	A	A	A	B	A	A	A	A	B	B	A	A	A	A	B	A

RIL families	57	58	59	61	62	63	64	66	67	68	69	70	71	72	74	75	77	78	79	80	81	82	83	84	85	86	87	88	89	90	91	92	93	94	95	96	97	98	99	101	103	104	105	106	107	108	109	111	112	114
AAFL_6_1	B	A	-	-	B	A	A	A	A	A	B	B	A	-	A	A	B	B	B	A	B	B	B	B	A	B	B	B	B	A	A	B	B	B	A	B	A	B	B	A	B	B	A	A	A	B	A	B	A	A
AKEZ_2_0	B	A	-	A	B	A	A	A	A	A	B	B	A	A	A	A	B	B	B	-	B	B	B	B	A	B	B	B	B	A	A	B	B	B	A	B	A	B	B	A	B	B	A	A	A	B	A	B	A	A
AQER_183_0	-	A	A	A	B	A	A	A	A	A	B	B	A	A	A	A	B	B	B	A	B	B	B	B	A	B	B	B	B	A	A	B	B	B	A	B	A	B	B	A	B	B	A	A	A	B	A	B	A	A
ATMV_35_0	-	A	-	A	B	A	A	A	A	A	B	B	A	A	A	A	B	B	B	A	B	B	B	B	A	B	B	B	B	A	A	B	B	B	A	B	A	B	B	A	B	B	A	A	A	B	A	B	A	A
BGOM_16_0	-	A	A	A	B	A	A	A	A	A	B	B	A	A	A	A	B	B	B	A	B	B	B	B	A	B	B	B	B	A	A	B	B	B	A	B	A	B	B	-	B	B	A	A	A	B	-	B	A	A
BGST_51_0	-	A	A	-	B	A	A	A	A	A	B	B	A	A	A	A	B	B	B	A	B	B	B	B	A	B	B	B	B	A	A	B	B	B	A	B	A	B	B	A	B	B	A	A	A	B	A	B	A	A

RIL families	115	116	117	118	119	120	121	122	123	125	127	128	130	235	236	237	238	239	240	242	243	244	245	247	248	249	250	251	252	253	254	255	256	257	258	259	260	263	264	265	266	267	268	269	270	271	275	276	278	280
AAFL_6_1	B	B	A	B	B	B	B	B	A	A	A	B	B	B	B	B	B	B	A	B	A	B	B	A	B	B	B	-	B	B	-	A	B	B	A	B	B	-	-	-	B	B	B	B	B	B	B	B	A	B
AKEZ_2_0	B	B	A	B	B	B	B	B	A	A	A	B	B	B	B	B	-	B	A	B	-	B	B	A	B	B	B	-	B	B	A	A	B	B	A	B	B	A	A	A	B	B	B	B	B	B	B	B	A	B
AQER_183_0	B	B	A	B	B	B	B	B	A	A	A	B	B	B	B	B	B	B	A	B	A	B	B	A	B	B	B	A	B	B	A	A	B	B	A	B	B	A	A	A	B	B	B	B	B	B	B	B	A	B
ATMV_35_0	B	B	A	B	B	B	B	B	A	A	A	B	B	B	B	B	B	B	A	B	A	B	B	A	B	B	B	A	B	B	A	A	B	B	A	B	B	A	A	A	B	B	B	B	B	B	B	B	A	B
BGOM_16_0	B	B	A	B	B	B	B	B	A	-	A	B	B	B	B	-	B	B	A	B	A	B	B	A	B	B	B	A	B	B	A	A	B	B	A	B	B	A	A	A	B	B	B	B	B	B	B	B	A	B
BGST_51_0	B	B	A	B	B	B	B	B	A	A	A	B	B	B	B	B	B	B	A	B	A	B	B	A	B	B	B	-	B	B	A	A	B	B	A	B	B	A	A	A	B	B	B	B	B	B	B	B	A	B

RIL families	281	283	284	286	287	289	290	291	292	294	296	298	299	300	301	302	303	304	305	306	307	312	314	315	316	317	318	319	321	322	325	326	327	328	329	330	332	333	334	336	338	339	340	341	343	344	345	346	348	349
AAFL_6_1	B	A	B	B	B	B	A	B	A	B	A	A	A	B	B	A	B	-	A	B	B	B	A	A	B	B	A	B	A	A	B	B	A	A	B	B	A	A	B	A	A	B	A	B	A	A	B	B	A	B
AKEZ_2_0	B	-	B	B	B	B	A	B	A	B	A	-	-	B	B	A	B	A	A	B	B	B	-	A	-	B	A	B	-	A	B	B	A	A	B	B	A	A	B	A	A	B	A	B	-	A	B	B	A	B
AQER_183_0	B	A	B	B	B	B	A	B	A	B	A	A	A	B	B	A	B	A	A	B	B	B	A	A	B	B	A	B	-	A	B	B	A	A	B	B	A	A	B	A	A	B	A	B	A	A	B	B	A	B
ATMV_35_0	B	A	B	B	B	B	A	B	A	B	A	A	A	B	B	A	B	A	A	B	B	B	A	A	B	B	A	B	A	A	B	B	A	A	B	B	A	A	B	A	A	B	A	B	A	A	B	B	A	B
BGOM_16_0	B	A	B	B	B	B	A	B	A	B	A	A	A	B	B	A	B	A	A	B	B	B	A	A	B	B	A	B	-	A	B	B	A	A	B	B	A	A	B	A	-	B	A	B	A	A	B	B	A	B
BGST_51_0	B	A	B	B	B	B	A	B	A	B	A	A	A	B	B	A	B	A	A	B	B	B	A	A	B	B	A	B	A	A	B	B	A	A	B	B	A	A	B	A	A	B	A	B	A	A	B	B	A	B

RIL families	350	353	354	358	360	362	364	365	366	368	369	370	371
AAFL_6_1	A	A	A	-	B	B	B	A	B	B	B	A	B
AKEZ_2_0	A	A	A	A	B	B	B	A	B	B	B	A	B
AQER_183_0	A	A	A	A	B	B	B	A	B	B	B	A	B
ATMV_35_0	A	A	A	A	B	B	B	A	B	B	B	A	B
BGOM_16_0	A	A	A	A	B	B	B	A	B	B	B	A	B
BGST_51_0	A	A	A	A	B	B	B	A	B	B	B	A	B

Figure S1 Haplotypes of all RIL families for the six molecular markers grouped in bin2 in linkage group 4. Alleles from the *L. sativa* parent are in red and alleles from the *L. serriola* parent are in blue. White represents missing data.

RIL families	1	2	3	4	5	6	7	8	9	11	12	13	14	15	16	17	18	19	20	21	22	23	24	25	26	27	29	30	31	32	33	34	35	36	37	39	40	41	42	43	44	45	47	48	50	51	52	53	55	56
ACAD_8_0	B	A	A	B	A	B	A	B	B	A	B	A	B	B	B	A	A	B	B	A	A	B	A	A	A	A	-	B	B	B	B	B	B	B	A	B	A	B	A	B	A	B	B	-	A	B	A	B	B	A
AKIS_7_0	B	A	-	B	A	B	A	B	B	A	B	A	B	B	B	A	A	B	B	A	A	B	A	A	A	A	B	B	B	B	B	B	B	B	A	B	A	B	A	B	A	B	B	A	A	B	A	B	B	A
ASDC_8_0	B	A	A	B	A	B	A	B	B	A	B	A	B	B	B	A	A	B	B	A	A	B	A	A	A	A	B	B	B	B	B	B	B	B	A	B	A	B	A	B	A	B	B	A	A	B	A	B	B	-
AXRS_1_0	B	A	A	B	A	B	A	B	B	-	B	A	B	B	B	A	A	B	B	A	A	B	A	A	A	A	B	B	B	B	B	B	B	B	A	B	-	B	A	B	A	B	B	A	A	B	A	B	B	A
AHPQ_36_0	B	A	A	B	A	B	A	B	B	A	B	A	B	B	B	A	A	B	B	A	A	B	A	A	A	A	B	B	B	B	B	B	B	B	A	B	A	B	A	B	A	B	B	A	A	B	A	B	B	A
AXWD_36_0	B	A	A	B	A	B	A	B	B	A	B	A	B	B	B	A	A	B	B	A	A	B	A	A	A	A	B	B	B	B	B	B	B	B	A	B	A	B	A	B	A	B	B	A	A	B	A	B	B	A

RIL families	57	58	59	61	62	63	64	66	67	68	69	70	71	72	74	75	77	78	79	80	81	82	83	84	85	86	87	88	89	90	91	92	93	94	95	96	97	98	99	101	103	104	105	106	107	108	109	111	112	114
ACAD_8_0	A	B	A	A	A	A	A	A	A	B	A	A	B	-	A	B	A	B	B	A	B	A	B	A	B	*B*	A	A	B	B	B	B	A	A	A	A	A	B	B	A	B	A	B	A	A	A	B	B	A	B
AKIS_7_0	A	B	A	-	A	A	A	A	A	B	A	A	B	A	A	B	A	B	B	A	B	A	B	A	B	-	-	A	B	B	B	B	A	A	A	A	A	B	B	A	B	A	B	A	A	A	B	B	A	B
ASDC_8_0	A	B	-	A	A	A	A	A	A	B	A	A	B	-	A	B	A	B	B	A	B	A	B	A	B	*A*	A	A	B	B	B	B	A	A	A	A	A	B	B	A	B	A	-	A	A	-	B	B	A	B
AXRS_1_0	A	B	A	A	A	A	A	A	A	B	A	-	B	A	A	B	A	B	B	-	B	A	B	-	B	*A*	A	A	B	B	B	B	A	A	A	-	A	B	B	A	B	A	B	A	A	A	B	B	A	B
AHPQ_36_0	A	B	A	A	A	A	A	A	A	B	A	A	B	A	A	B	A	B	B	A	B	A	B	A	B	*A*	A	A	B	B	B	B	A	A	A	A	A	B	B	A	B	A	-	A	A	A	B	B	A	B
AXWD_36_0	A	B	A	A	A	A	A	A	A	B	A	A	B	A	A	B	A	B	B	A	B	A	B	A	B	*A*	A	A	B	B	B	B	A	A	A	A	A	B	B	A	B	A	-	A	A	A	B	B	A	B

RIL families	115	116	117	118	119	120	121	122	123	125	127	128	130	235	236	237	238	239	240	242	243	244	245	247	248	249	250	251	252	253	254	255	256	257	258	259	260	263	264	265	266	267	268	269	270	271	275	276	278	280
ACAD_8_0	A	A	A	B	A	A	A	A	A	A	B	A	A	A	A	A	A	A	A	A	B	B	A	B	A	B	A	B	B	B	A	A	B	B	B	A	A	A	B	B	B	A	A	B	B	B	B	B	B	B
AKIS_7_0	A	A	A	B	A	A	A	A	A	A	B	A	A	A	A	A	A	A	A	A	B	B	A	B	A	B	A	B	B	B	A	A	B	B	B	A	A	A	B	B	B	A	-	B	B	B	B	B	B	B
ASDC_8_0	A	A	A	B	A	A	A	A	-	-	B	A	A	A	A	A	A	-	A	A	B	B	A	B	A	B	A	B	B	B	A	A	B	B	B	A	A	A	B	B	B	A	A	B	B	B	B	B	B	B
AXRS_1_0	A	A	A	B	A	A	A	-	-	A	B	-	A	A	A	A	A	A	A	A	B	B	-	B	A	B	A	B	B	B	A	A	B	B	B	A	A	A	B	B	B	A	A	B	B	B	B	B	B	B
AHPQ_36_0	A	A	A	B	A	A	A	A	A	A	B	A	A	A	A	A	A	A	A	A	B	B	A	B	A	B	A	B	B	B	A	A	B	B	B	A	A	A	B	B	B	A	A	B	B	B	B	B	B	B
AXWD_36_0	A	A	A	B	A	A	A	A	A	A	B	A	A	A	A	A	A	A	A	A	B	B	A	B	A	B	A	B	B	B	A	A	B	B	B	A	A	A	B	B	B	A	A	B	B	B	B	B	B	B

RIL families	281	283	284	286	287	289	290	291	292	294	296	298	299	300	301	302	303	304	305	306	307	312	314	315	316	317	318	319	321	322	325	326	327	328	329	330	332	333	334	336	338	339	340	341	343	344	345	346	348	349
ACAD_8_0	A	B	B	B	B	B	A	B	B	B	A	A	A	B	B	B	B	B	B	B	B	A	B	A	A	A	A	A	B	A	B	B	B	B	B	B	B	-	A	B	A	B	A	A	B	B	B	B	B	B
AKIS_7_0	A	B	B	B	B	B	A	B	B	B	A	A	A	B	B	B	B	B	B	B	B	A	B	A	A	A	A	A	B	A	B	B	B	B	B	B	B	*B*	A	B	A	B	A	A	B	B	B	B	B	B
ASDC_8_0	A	B	B	B	B	B	A	B	B	B	A	A	A	B	B	B	B	B	B	B	B	A	B	A	A	A	A	A	B	A	B	B	B	B	B	B	B	-	A	B	A	B	A	A	B	B	B	B	B	B
AXRS_1_0	A	B	B	B	B	B	A	B	B	B	A	A	A	B	B	B	B	B	B	B	B	A	B	A	A	A	-	A	B	A	B	B	B	B	B	B	B	*A*	A	B	A	B	A	A	B	B	B	B	B	B
AHPQ_36_0	A	B	B	B	B	B	A	B	B	B	A	A	A	B	B	B	B	B	B	B	B	A	B	A	A	A	A	A	B	A	B	B	B	B	B	B	B	-	A	B	A	B	A	A	B	B	B	B	B	B
AXWD_36_0	A	B	B	B	B	B	A	B	B	B	A	A	A	B	B	B	B	B	B	B	B	A	B	A	A	A	A	A	B	A	B	B	B	B	B	B	B	-	A	B	A	B	A	A	B	B	B	B	B	B

RIL families	350	353	354	358	360	362	364	365	366	368	369	370	371
ACAD_8_0	B	B	B	A	A	B	B	B	A	A	B	B	B
AKIS_7_0	B	B	B	A	A	B	B	B	A	A	B	B	B
ASDC_8_0	B	B	B	A	A	B	B	B	A	A	B	B	B
AXRS_1_0	B	B	B	A	-	B	B	B	A	A	B	B	B
AHPQ_36_0	B	B	B	A	A	B	B	B	A	A	B	B	B
AXWD_36_0	B	B	B	A	A	B	B	B	A	A	B	B	B

Figure S2 Haplotypes of all RIL families for the six molecular markers grouped in sbin36 in linkage group 4. RIL individuals 86 and 333 (highlighted in yellow) show a mix of calls for the markers. Alleles from the *L. sativa* parent are in red and the alleles from the *L. serriola* parent are in blue. White represents missing data.

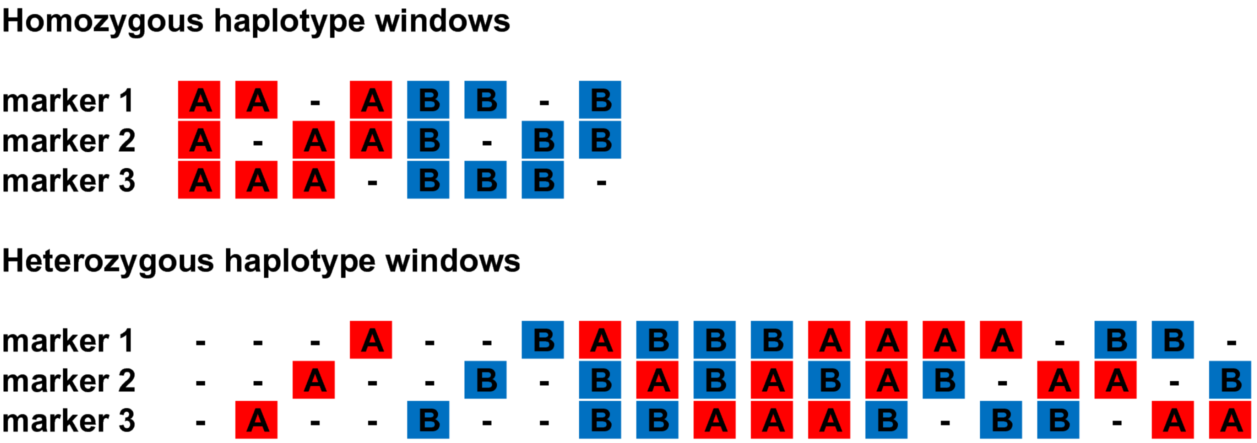

Figure S3 Homozygous or heterozygous haplotypes identified using a sliding window of three markers. Alleles from the *L. sativa* and *L. serriola* parents are in red and blue respectively. - = missing data.

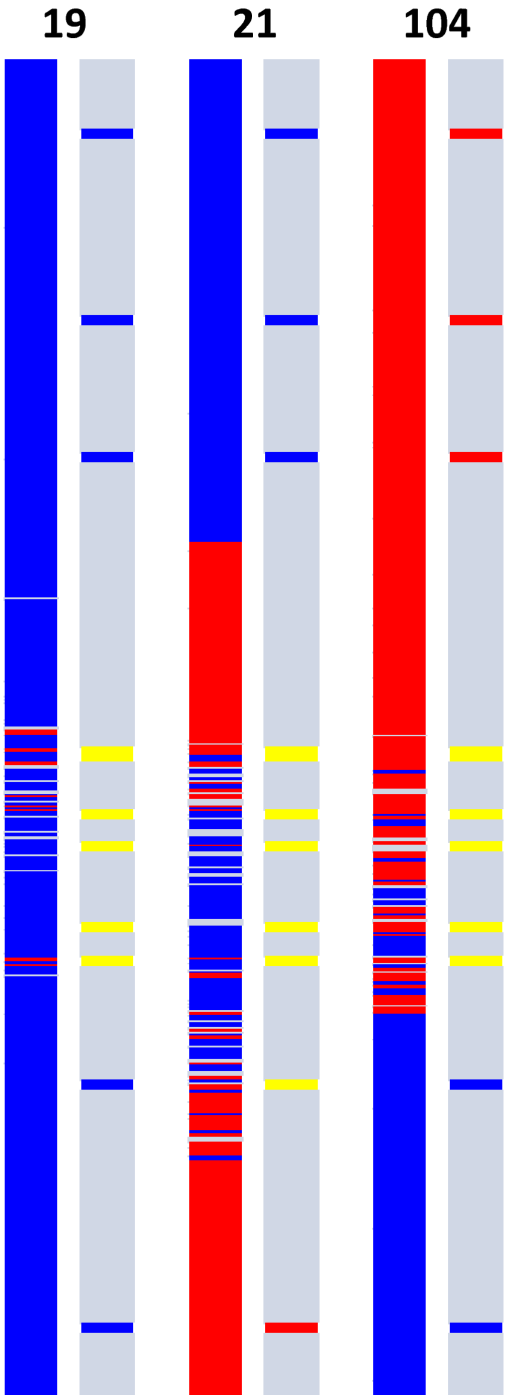

Figure S4 Correspondence of heterozygous regions of LG1 in three RILs (#19, 21, and 104) estimated using the GeneChip (left) and using Illumina Golden Gate SNP markers (right). Genotype calls are indicated as: red, *L. sativa* allele; blue, *L. serriola* allele; yellow, heterozygous SNP; and white, no allele called.

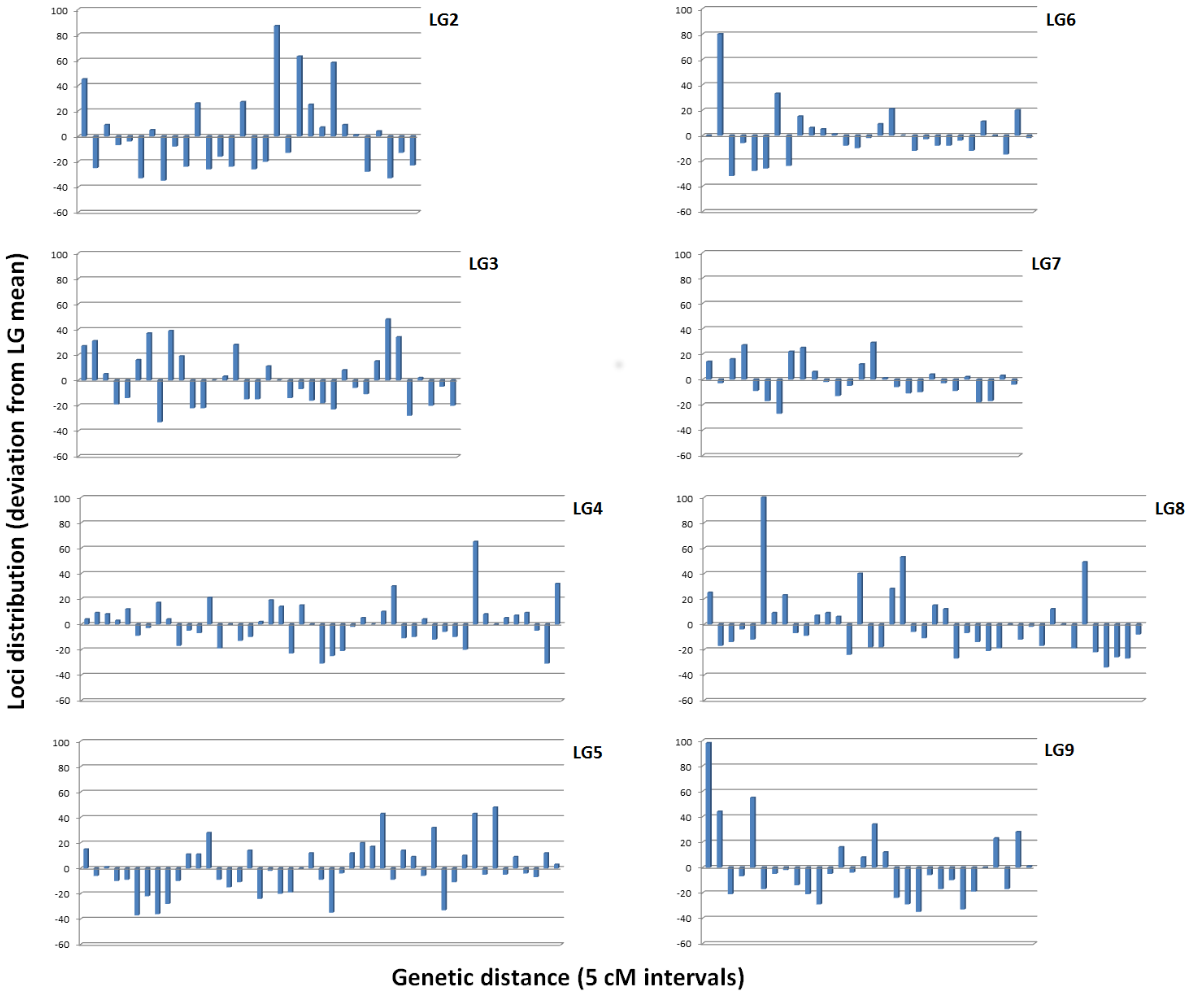

Figure S5 Average distribution of loci in intervals of 5 cM along LG2 to LG9. Deviations from the mean value for LG2 (x=53), LG3 (x=40), LG4 (x=40), LG5 (x=43), LG6 (x=42), LG7 (x=45), LG8 (x=48) and LG9 (x=38) is shown by bars.

Table S1 The 52 accessions of *L. sativa* and *L. serriola* used to assess the distribution of genetic diversity along each linkage group.

Expt. ID	Accession name	Species	Lettuce type	Group[a]
BSP001	Cantabria	*L. sativa*	butterhead	butterhead
BSP002	Marianna	*L. sativa*	butterhead	butterhead
BSP003	Nadine	*L. sativa*	butterhead	butterhead
BSP004	Rex	*L. sativa*	butterhead	butterhead
BSP005	Fenston	*L. sativa*	butterhead	butterhead
UCD021	Diana	*L. sativa*	butterhead	butterhead
UCD008	Olof	*L. sativa*	butterhead	butterhead
UCD005	Kordaat	*L. sativa*	butterhead	butterhead
UCD007	Mariska	*L. sativa*	butterhead	butterhead
BSP006	Yael	*L. sativa*	romaine	romaine
BSP007	Filipus	*L. sativa*	romaine	romaine
BSP008	Odra	*L. sativa*	romaine	romaine
BSP009	Pinokkio	*L. sativa*	romaine	romaine
BSP010	Romaserra	*L. sativa*	romaine	romaine
UCD002	Valmaine	*L. sativa*	romaine	romaine
BSP011	Angie	*L. sativa*	crisphead - batavia green	crisphead
BSP012	Reglice	*L. sativa*	crisphead - batavia green	crisphead
BSP013	Funly	*L. sativa*	crisphead - batavia green	crisphead
BSP014	Noisette	*L. sativa*	crisphead - batavia green	crisphead
BSP015	Iceberg	*L. sativa*	crisphead - batavia red	crisphead
BSP016	Luana	*L. sativa*	crisphead - batavia red	crisphead
BSP017	Astral	*L. sativa*	crisphead - iceberg	crisphead
BSP018	Tiger	*L. sativa*	crisphead - iceberg	crisphead
BSP019	Robinson	*L. sativa*	crisphead - iceberg	crisphead
BSP020	Lilach	*L. sativa*	crisphead - iceberg	crisphead
UCD022	Greenlake	*L. sativa*	crisphead-iceberg	crisphead
UCD001	Salinas	*L. sativa*	crisphead - iceberg	crisphead
UCD004	Calmar	*L. sativa*	crisphead - iceberg	crisphead
UCD006	Vanguard 75	*L. sativa*	crisphead - iceberg	crisphead
BSP021	Berwick	*L. sativa*	curly	leafy
BSP022	Cancan	*L. sativa*	frisee	leafy
BSP023	Xanadu	*L. sativa*	grasse	leafy

BSP024	Aído	*L. sativa*	grasse red	leafy
BSP025	Xena	*L. sativa*	green leaf	leafy
UCD003	Salad Bowl	*L. sativa*	green leaf	leafy
BSP026	Deep Red	*L. sativa*	red leaf	leafy
BSP027	Locarno	*L. sativa*	lollo bionda	leafy
BSP028	Concorde	*L. sativa*	lollo rossa	leafy
BSP029	Pareo	*L. sativa*	green oakleaf	leafy
BSP030	Grenadine	*L. sativa*	red oakleaf	leafy
UCD014	PI251246	*L. sativa*	oil	leafy
UCD009	UC96US23	*L. serriola*	wild	*L. sativa* vs. *L. serriola*
UCD010	LSE18	*L. serriola*	wild	*L. sativa* vs. *L. serriola*
UCD011	LS102	*L. serriola*	wild	*L. sativa* vs. *L. serriola*
UCD012	CGN14263	*L. serriola*	wild	*L. sativa* vs. *L. serriola*
UCD013	CGN14278	*L. serriola*	wild	*L. sativa* vs. *L. serriola*
UCD016	UC1	*L. saligna*	wild	
UCD017	PI491204	*L. saligna*	wild	
UCD015	CGN5271	*L. saligna*	wild	
UCD018	UK-1	*L. virosa*	wild	
UCD019	PIVT208	*L. virosa*	wild	
UCD020	PI274378	*L. perennis*	wild	

[a] Group designation used in Figure 8.
