# Supplementary material for: An Ultra-High-Density, Transcript-Based, Genetic Map of Lettuce
Source: G3 (Bethesda). 2013 Apr 1;3(4):617–31. doi: 10.1534/g3.112.004929 (PMC3618349; doi:10.1534/g3.112.004929)
Supplement: Supporting Information [file supp_g3.112.004929_FigureS1.pdf]

RIL families	1	2	3	4	5	6	7	8	9	11	12	13	14	15	16	17	18	19	20	21	22	23	24	25	26	27	29	30	31	32	33	34	35	36	37	39	40	41	42	43	44	45	47	48	50	51	52	53	55	56
AAFL_6_1	B	B	B	A	A	A	B	B	B	B	A	B	B	A	A	B	B	B	B	B	A	A	A	B	B	A	A	B	A	B	A	A	B	A	A	A	A	B	A	A	A	A	B	B	A	A	A	A	B	A
AKEZ_2_0	B	B	B	A	A	A	B	B	B	B	A	B	B	A	A	B	B	B	B	B	A	A	A	B	B	A	A	B	A	B	A	A	B	A	A	A	A	B	A	A	A	A	B	B	A	A	A	A	B	A
AQER_183_0	B	B	B	A	A	A	B	B	B	B	A	B	B	A	A	B	B	B	B	B	A	A	A	B	B	A	A	B	A	B	A	A	B	A	A	A	A	B	A	A	A	A	B	B	A	A	A	A	B	A
ATMV_35_0	B	B	B	A	A	A	B	B	B	B	A	B	B	A	-	B	B	B	B	B	A	A	A	B	B	A	A	B	A	B	A	A	B	A	A	A	A	B	A	A	A	A	B	B	A	A	A	A	B	A
BGOM_16_0	B	B	B	A	A	A	B	B	B	B	A	B	B	A	A	B	B	B	B	B	-	A	A	B	B	A	A	B	A	B	A	A	B	A	A	A	A	B	A	A	A	A	B	B	A	A	A	A	B	A
BGST_51_0	B	B	B	A	A	A	B	B	B	B	A	B	B	A	A	B	B	B	B	B	A	A	A	B	B	A	A	B	A	B	A	A	B	A	A	A	A	B	A	A	A	A	B	B	A	A	A	A	B	A

RIL families	57	58	59	61	62	63	64	66	67	68	69	70	71	72	74	75	77	78	79	80	81	82	83	84	85	86	87	88	89	90	91	92	93	94	95	96	97	98	99	101	103	104	105	106	107	108	109	111	112	114
AAFL_6_1	B	A	-	-	B	A	A	A	A	A	B	B	A	-	A	A	B	B	B	A	B	B	B	B	A	B	B	B	B	A	A	B	B	B	A	B	A	B	B	A	B	B	A	A	A	B	A	B	A	A
AKEZ_2_0	B	A	-	A	B	A	A	A	A	A	B	B	A	A	A	A	B	B	B	-	B	B	B	B	A	B	B	B	B	A	A	B	B	B	A	B	A	B	B	A	B	B	A	A	A	B	A	B	A	A
AQER_183_0	-	A	A	A	B	A	A	A	A	A	B	B	A	A	A	A	B	B	B	A	B	B	B	B	A	B	B	B	B	A	A	B	B	B	A	B	A	B	B	A	B	B	A	A	A	B	A	B	A	A
ATMV_35_0	-	A	-	A	B	A	A	A	A	A	B	B	A	A	A	A	B	B	B	A	B	B	B	B	A	B	B	B	B	A	A	B	B	B	A	B	A	B	B	A	B	B	A	A	A	B	A	B	A	A
BGOM_16_0	-	A	A	A	B	A	A	A	A	A	B	B	A	A	A	A	B	B	B	A	B	B	B	B	A	B	B	B	B	A	A	B	B	B	A	B	A	B	B	-	B	B	A	A	A	B	-	B	A	A
BGST_51_0	-	A	A	-	B	A	A	A	A	A	B	B	A	A	A	A	B	B	B	A	B	B	B	B	A	B	B	B	B	A	A	B	B	B	A	B	A	B	B	A	B	B	A	A	A	B	A	B	A	A

RIL families	115	116	117	118	119	120	121	122	123	125	127	128	130	235	236	237	238	239	240	242	243	244	245	247	248	249	250	251	252	253	254	255	256	257	258	259	260	263	264	265	266	267	268	269	270	271	275	276	278	280
AAFL_6_1	B	B	A	B	B	B	B	B	A	A	A	B	B	B	B	B	B	B	A	B	A	B	B	A	B	B	B	-	B	B	-	A	B	B	A	B	B	-	-	-	B	B	B	B	B	B	B	B	A	B
AKEZ_2_0	B	B	A	B	B	B	B	B	A	A	A	B	B	B	B	B	-	B	A	B	-	B	B	A	B	B	B	-	B	B	A	A	B	B	A	B	B	A	A	A	B	B	B	B	B	B	B	B	A	B
AQER_183_0	B	B	A	B	B	B	B	B	A	A	A	B	B	B	B	B	B	B	A	B	A	B	B	A	B	B	B	A	B	B	A	A	B	B	A	B	B	A	A	A	B	B	B	B	B	B	B	B	A	B
ATMV_35_0	B	B	A	B	B	B	B	B	A	A	A	B	B	B	B	B	B	B	A	B	A	B	B	A	B	B	B	A	B	B	A	A	B	B	A	B	B	A	A	A	B	B	B	B	B	B	B	B	A	B
BGOM_16_0	B	B	A	B	B	B	B	B	A	-	A	B	B	B	B	-	B	B	A	B	A	B	B	A	B	B	B	A	B	B	A	A	B	B	A	B	B	A	A	A	B	B	B	B	B	B	B	B	A	B
BGST_51_0	B	B	A	B	B	B	B	B	A	A	A	B	B	B	B	B	B	B	A	B	A	B	B	A	B	B	B	-	B	B	A	A	B	B	A	B	B	A	A	A	B	B	B	B	B	B	B	B	A	B

RIL families	281	283	284	286	287	289	290	291	292	294	296	298	299	300	301	302	303	304	305	306	307	312	314	315	316	317	318	319	321	322	325	326	327	328	329	330	332	333	334	336	338	339	340	341	343	344	345	346	348	349
AAFL_6_1	B	A	B	B	B	B	A	B	A	B	A	A	A	B	B	A	B	-	A	B	B	B	A	A	B	B	A	B	A	A	B	B	A	A	B	B	A	A	B	A	A	B	A	B	A	A	B	B	A	B
AKEZ_2_0	B	-	B	B	B	B	A	B	A	B	A	-	-	B	B	A	B	A	A	B	B	B	-	A	-	B	A	B	-	A	B	B	A	A	B	B	A	A	B	A	A	B	A	B	-	A	B	B	A	B
AQER_183_0	B	A	B	B	B	B	A	B	A	B	A	A	A	B	B	A	B	A	A	B	B	B	A	A	B	B	A	B	-	A	B	B	A	A	B	B	A	A	B	A	A	B	A	B	A	A	B	B	A	B
ATMV_35_0	B	A	B	B	B	B	A	B	A	B	A	A	A	B	B	A	B	A	A	B	B	B	A	A	B	B	A	B	A	A	B	B	A	A	B	B	A	A	B	A	A	B	A	B	A	A	B	B	A	B
BGOM_16_0	B	A	B	B	B	B	A	B	A	B	A	A	A	B	B	A	B	A	A	B	B	B	A	A	B	B	A	B	-	A	B	B	A	A	B	B	A	A	B	A	-	B	A	B	A	A	B	B	A	B
BGST_51_0	B	A	B	B	B	B	A	B	A	B	A	A	A	B	B	A	B	A	A	B	B	B	A	A	B	B	A	B	A	A	B	B	A	A	B	B	A	A	B	A	A	B	A	B	A	A	B	B	A	B

RIL families	350	353	354	358	360	362	364	365	366	368	369	370	371
AAFL_6_1	A	A	A	-	B	B	B	A	B	B	B	A	B
AKEZ_2_0	A	A	A	A	B	B	B	A	B	B	B	A	B
AQER_183_0	A	A	A	A	B	B	B	A	B	B	B	A	B
ATMV_35_0	A	A	A	A	B	B	B	A	B	B	B	A	B
BGOM_16_0	A	A	A	A	B	B	B	A	B	B	B	A	B
BGST_51_0	A	A	A	A	B	B	B	A	B	B	B	A	B

Figure S1 Haplotypes of all RIL families for the six molecular markers grouped in bin2 in linkage group 4. Alleles from the *L. sativa* parent are in red and alleles from the *L. serriola* parent are in blue. White represents missing data.
