# Supplementary figures and images for: An Ultra-High-Density, Transcript-Based, Genetic Map of Lettuce
Source: G3 (Bethesda). 2013 Apr 1;3(4):617–31. doi: 10.1534/g3.112.004929 (PMC3618349; doi:10.1534/g3.112.004929)

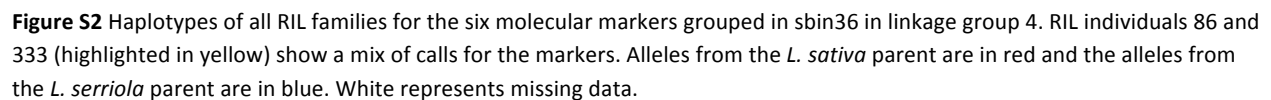

Supplement: Supporting Information [file supp_g3.112.004929_FigureS2.pdf]
