# Supplementary material for: An Ultra-High-Density, Transcript-Based, Genetic Map of Lettuce
Source: G3 (Bethesda). 2013 Apr 1;3(4):617–31. doi: 10.1534/g3.112.004929 (PMC3618349; doi:10.1534/g3.112.004929)
Supplement: Supporting Information [file supp_g3.112.004929_FigureS3.pdf]

Homozygous haplotype windows

marker 1	A	A	-	A	B	B	-	B
marker 2	A	-	A	A	B	-	B	B
marker 3	A	A	A	-	B	B	B	-

Heterozygous haplotype windows

marker 1	-	-	-	A	-	-	B	A	B	B	B	A	A	A	A	-	B	B	-
marker 2	-	-	A	-	-	B	-	B	A	B	A	B	A	B	-	A	A	-	B
marker 3	-	A	-	-	B	-	-	B	B	A	A	A	B	-	B	B	-	A	A

Figure S3 Homozygous or heterozygous haplotypes identified using a sliding window of three markers. Alleles from the *L. sativa* and *L. serriola* parents are in red and blue respectively. - = missing data.
