# Supplementary material for: An Ultra-High-Density, Transcript-Based, Genetic Map of Lettuce
Source: G3 (Bethesda). 2013 Apr 1;3(4):617–31. doi: 10.1534/g3.112.004929 (PMC3618349; doi:10.1534/g3.112.004929)
Supplement: Supporting Information [file supp_g3.112.004929_FigureS4.pdf]

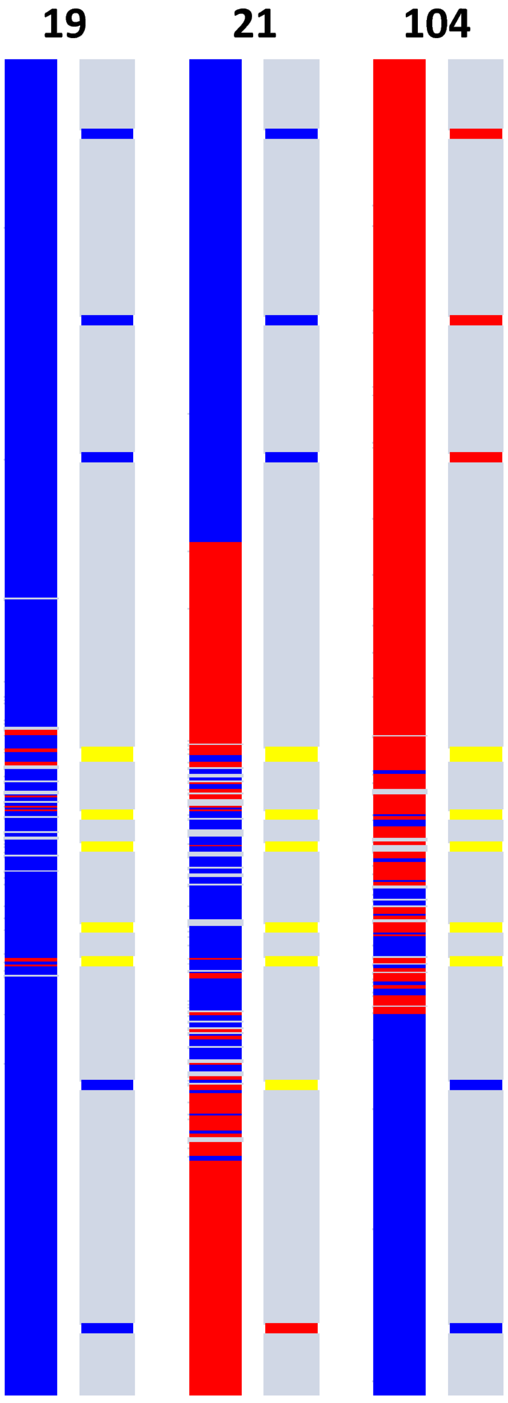

Figure S4 Correspondence of heterozygous regions of LG1 in three RILs (#19, 21, and 104) estimated using the GeneChip (left) and using Illumina Golden Gate SNP markers (right). Genotype calls are indicated as: red, *L. sativa* allele; blue, *L. serriola* allele; yellow, heterozygous SNP; and white, no allele called.
