# Supplementary material for: An Ultra-High-Density, Transcript-Based, Genetic Map of Lettuce
Source: G3 (Bethesda). 2013 Apr 1;3(4):617–31. doi: 10.1534/g3.112.004929 (PMC3618349; doi:10.1534/g3.112.004929)
Supplement: Supporting Information [file supp_g3.112.004929_FigureS5.pdf]

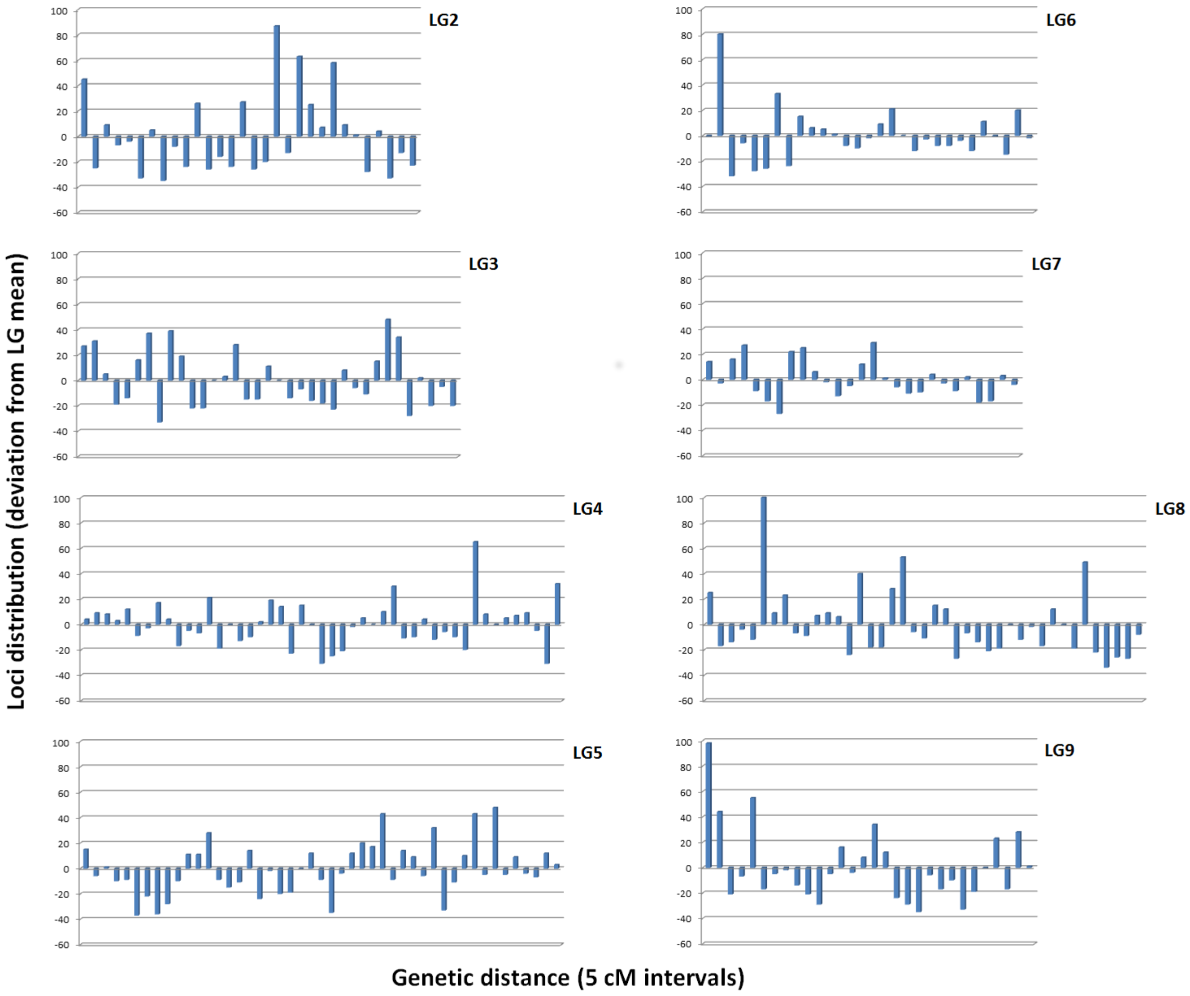

Figure S5 Average distribution of loci in intervals of 5 cM along LG2 to LG9. Deviations from the mean value for LG2 (x=53), LG3 (x=40), LG4 (x=40), LG5 (x=43), LG6 (x=42), LG7 (x=45), LG8 (x=48) and LG9 (x=38) is shown by bars.
