# Supplementary material for: An Ultra-High-Density, Transcript-Based, Genetic Map of Lettuce
Source: G3 (Bethesda). 2013 Apr 1;3(4):617–31. doi: 10.1534/g3.112.004929 (PMC3618349; doi:10.1534/g3.112.004929)
Supplement: Supporting Information [file supp_g3.112.004929_TableS1.pdf]

Table S1 The 52 accessions of *L. sativa* and *L. serriola* used to assess the distribution of genetic diversity along each linkage group.

Expt. ID	Accession name	Species	Lettuce type	Group[a]
BSP001	Cantabria	*L. sativa*	butterhead	butterhead
BSP002	Marianna	*L. sativa*	butterhead	butterhead
BSP003	Nadine	*L. sativa*	butterhead	butterhead
BSP004	Rex	*L. sativa*	butterhead	butterhead
BSP005	Fenston	*L. sativa*	butterhead	butterhead
UCD021	Diana	*L. sativa*	butterhead	butterhead
UCD008	Olof	*L. sativa*	butterhead	butterhead
UCD005	Kordaat	*L. sativa*	butterhead	butterhead
UCD007	Mariska	*L. sativa*	butterhead	butterhead
BSP006	Yael	*L. sativa*	romaine	romaine
BSP007	Filipus	*L. sativa*	romaine	romaine
BSP008	Odra	*L. sativa*	romaine	romaine
BSP009	Pinokkio	*L. sativa*	romaine	romaine
BSP010	Romaserra	*L. sativa*	romaine	romaine
UCD002	Valmaine	*L. sativa*	romaine	romaine
BSP011	Angie	*L. sativa*	crisphead - batavia green	crisphead
BSP012	Reglice	*L. sativa*	crisphead - batavia green	crisphead
BSP013	Funly	*L. sativa*	crisphead - batavia green	crisphead
BSP014	Noisette	*L. sativa*	crisphead - batavia green	crisphead
BSP015	Iceberg	*L. sativa*	crisphead - batavia red	crisphead
BSP016	Luana	*L. sativa*	crisphead - batavia red	crisphead
BSP017	Astral	*L. sativa*	crisphead - iceberg	crisphead
BSP018	Tiger	*L. sativa*	crisphead - iceberg	crisphead
BSP019	Robinson	*L. sativa*	crisphead - iceberg	crisphead
BSP020	Lilach	*L. sativa*	crisphead - iceberg	crisphead
UCD022	Greenlake	*L. sativa*	crisphead-iceberg	crisphead
UCD001	Salinas	*L. sativa*	crisphead - iceberg	crisphead
UCD004	Calmar	*L. sativa*	crisphead - iceberg	crisphead
UCD006	Vanguard 75	*L. sativa*	crisphead - iceberg	crisphead
BSP021	Berwick	*L. sativa*	curly	leafy
BSP022	Cancan	*L. sativa*	frisee	leafy
BSP023	Xanadu	*L. sativa*	grasse	leafy

BSP024	Aído	*L. sativa*	grasse red	leafy
BSP025	Xena	*L. sativa*	green leaf	leafy
UCD003	Salad Bowl	*L. sativa*	green leaf	leafy
BSP026	Deep Red	*L. sativa*	red leaf	leafy
BSP027	Locarno	*L. sativa*	lollo bionda	leafy
BSP028	Concorde	*L. sativa*	lollo rossa	leafy
BSP029	Pareo	*L. sativa*	green oakleaf	leafy
BSP030	Grenadine	*L. sativa*	red oakleaf	leafy
UCD014	PI251246	*L. sativa*	oil	leafy
UCD009	UC96US23	*L. serriola*	wild	*L. sativa* vs. *L. serriola*
UCD010	LSE18	*L. serriola*	wild	*L. sativa* vs. *L. serriola*
UCD011	LS102	*L. serriola*	wild	*L. sativa* vs. *L. serriola*
UCD012	CGN14263	*L. serriola*	wild	*L. sativa* vs. *L. serriola*
UCD013	CGN14278	*L. serriola*	wild	*L. sativa* vs. *L. serriola*
UCD016	UC1	*L. saligna*	wild	
UCD017	PI491204	*L. saligna*	wild	
UCD015	CGN5271	*L. saligna*	wild	
UCD018	UK-1	*L. virosa*	wild	
UCD019	PIVT208	*L. virosa*	wild	
UCD020	PI274378	*L. perennis*	wild	

[a] Group designation used in Figure 8.
